# Exploratory clinical trial on the safety and bactericidal effect of 222-nm ultraviolet C irradiation in healthy humans

**DOI:** 10.1371/journal.pone.0235948

**Published:** 2020-08-12

**Authors:** Tomoaki Fukui, Takahiro Niikura, Takahiro Oda, Yohei Kumabe, Hiroyuki Ohashi, Masahiro Sasaki, Tatsushi Igarashi, Makoto Kunisada, Nozomi Yamano, Keisuke Oe, Tomoyuki Matsumoto, Takehiko Matsushita, Shinya Hayashi, Chikako Nishigori, Ryosuke Kuroda

**Affiliations:** 1 Department of Orthopaedic Surgery, Kobe University Graduate School of Medicine, Kobe, Hyogo, Japan; 2 Ushio Inc., Chiyoda-ku, Tokyo, Japan; 3 Division of Dermatology, Department of Internal Related, Kobe University Graduate School of Medicine, Kobe, Hyogo, Japan; Universidade do Extremo Sul Catarinense, BRAZIL

## Abstract

**Introduction:**

Surgical site infection is one of the most severe complications of surgical treatments. However, the optimal procedure to prevent such infections remains uninvestigated. Ultraviolet radiation C (UVC) with a short wavelength has a high bactericidal effect; however, it is cytotoxic. Nonetheless, given that UVC with a wavelength of 222 nm reaches only the stratum corneum, it does not affect the skin cells. This study aimed to investigate the safety of 222-nm UVC irradiation and to examine its skin sterilization effect in healthy volunteers.

**Methods:**

This trial was conducted on 20 healthy volunteers. The back of the subject was irradiated with 222-nm UVC at 50–500 mJ/cm^2^, and the induced erythema (redness of skin) was evaluated. Subsequently, the back was irradiated with a maximum amount of UVC not causing erythema, and the skin swabs before and after the irradiation were cultured. The number of colonies formed after 24 hours was measured. In addition, cyclobutene pyrimidine dimer (CPD) as an indicator of DNA damage was measured using skin tissues of the nonirradiated and irradiated regions.

**Results:**

All subjects experienced no erythema at all doses. The back of the subject was irradiated at 500 mJ/cm^2^, and the number of bacterial colonies in the skin swab culture was significantly decreased by 222-nm UVC irradiation. The CPD amount produced in the irradiated region was slightly but significantly higher than that of the non-irradiated region.

**Conclusion:**

A 222-nm UVC at 500 mJ/cm^2^ was a safe irradiation dose and possessed bactericidal effects. In the future, 222-nm UVC irradiation is expected to contribute to the prevention of perioperative infection.

## Introduction

Surgical treatment has the risk of some complications, and surgical site infection (SSI) is recognized as one of the most serious complications. Once the infection is established, the treatment becomes complicated, which imposes a heavy burden on both the patient and medical staff. For example, postoperative infection after osteosynthesis for bone fracture occasionally leads to osteomyelitis, and treatment could take several years, resulting in limb amputation in worst cases. Although administration of antibiotics based on the established guidelines is routinely used to prevent SSI, SSI still remains a frequent occurrence. Therefore, it is necessary to establish more effective precautionary measures [1].

There are two kinds of infection route for bacteria causing SSI. One is endogenous substances, such as resident bacteria on the patient's skin and nasal mucosa, and the other is exogenous bacteria, such as airborne bacteria [2, 3]. Although there is no consensus on which is more important currently, many reports emphasize the involvement of endogenous bacterial infection [4, 5]. Therefore, prevention of endogenous bacterial infection might be considered as the basis [6]. Incise drape is widely used and effective for preventing SSI; however, it could get removed during surgery, increasing the infection rate owing to endogenous bacterial breeding around the incision [7]. To suppress both endogenous and exogenous bacterial infections, it is necessary to continuously suppress the contamination of the surgical wound due to breeding of intrinsic skin bacteria during surgery, even in surgical fields sufficiently disinfected before surgery.

Ultraviolet radiation is classified into three bands as follows: ultraviolet radiation A (UVA), ultraviolet radiation B (UVB), and ultraviolet radiation C (UVC) based on the wavelength, depending on its biological effect. UVC has a short wavelength ranging from 200 to 280 nm and has a high DNA absorption coefficient; it has a high bactericidal effect [8]. A previous report demonstrated that intraoperative irradiation of 254-nm UVC from an irradiator installed on the ceiling of an operation room decreased the postoperative infection rate [9]. Currently, devices used for irradiation of 254-nm UVC are approved for the treatment of infected wounds in the United States and Canada and are clinically used [10–12]. However, irradiation with UVC is cytotoxic, and there is a risk of developing malignant tumor; thus, a safer way is desirable.

Upon exposure to the skin, 254-nm UVC passes through the stratum corneum and reaches the epidermis, affecting the epidermal cells. On the contrary, 222-nm UVC has a high protein absorption coefficient [13] and reaches only the outermost stratum corneum of the epidermis [14]. Consequently, it does not affect the skin cells; therefore, it has a possibility of being a safer method of UVC irradiation theoretically.

Supposedly, irradiation with 222-nm UVC may dramatically reduce the SSI rate and can be clinically applied to the surgical field. However, to our knowledge, although the safety and sterilizing effect of this type of irradiation were demonstrated in an animal study [15], these have never been studied in humans. Therefore, this study aimed to investigate the feasibility and potential bactericidal effect of this type of irradiation, which is a prerequisite for future clinical trials on the prevention of SSIs and avoidable perioperative complications.

## Methods

### Study design

This study is a single arm exploratory clinical trial involving healthy subjects conducted in Kobe University Hospital. The protocol and informed consent form of this study were approved by the Institutional Review Board of Kobe University Hospital (approval number 290014). This study is registered with the University Hospital Medical Information Network (UMIN) Clinical Trial Registry (UMIN000027449). The trial office cited is in the Department of Orthopaedic Surgery, Kobe University Hospital.

### Participants

**Fig 1:**
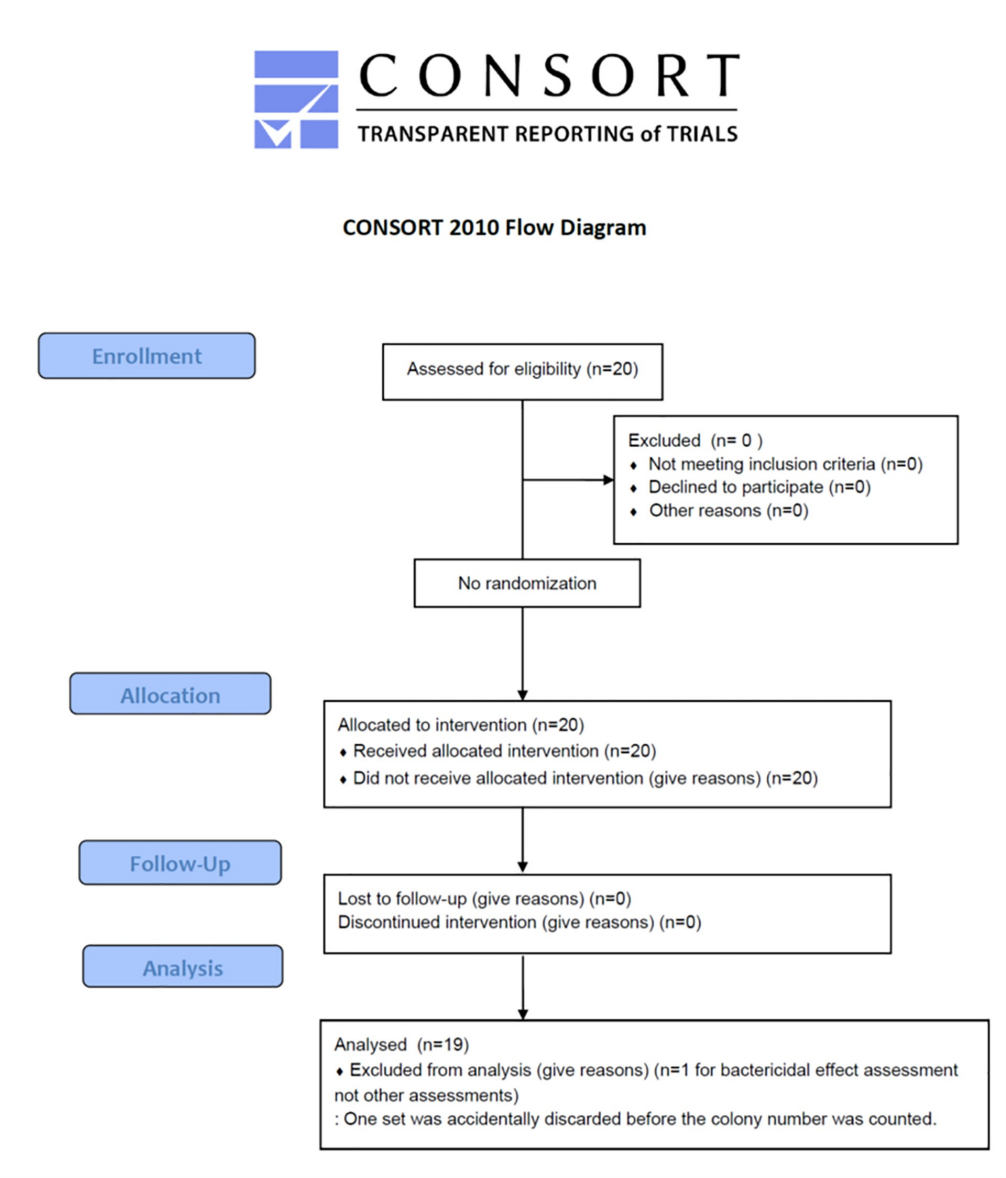
CONSORT diagram describing participant flow through the study.

This study enrolled healthy volunteers aged 20–80 years with no abnormality at the site of UVC irradiation, who provided written consent for participation, and did not meet any of the exclusion criteria listed in Table 1. Period for participant recruitment was set as 11 months from August 25th 2017, and the follow-up period was set for three months after the last irradiation. All 20 enrolled volunteers were men with a mean age of 33.7 (26–43) years. Fig 1 provides the CONSORT diagram for participant flow through the study.

**Table 1:** Exclusion criteria.

1) Sensitivity to drugs, such as allergies.
2) Abnormalities at the site of UVC irradiation on the back, such as dermatitis or trauma.
3) Regular use of topical medication or patches at the site of UVC irradiation on the back.
4) Tattoos at the site of UVC irradiation on the back.
5) Pregnancy or possibility of pregnancy or breast-feeding.
6) Participation in other clinical trials within 4 months.
7) Rejection as inappropriate for participation by the physician in charge of the clinical trial.

Abbreviation: UVC, ultraviolet radiation C.

### Procedures

#### UVC irradiation equipment

**Fig 2:**
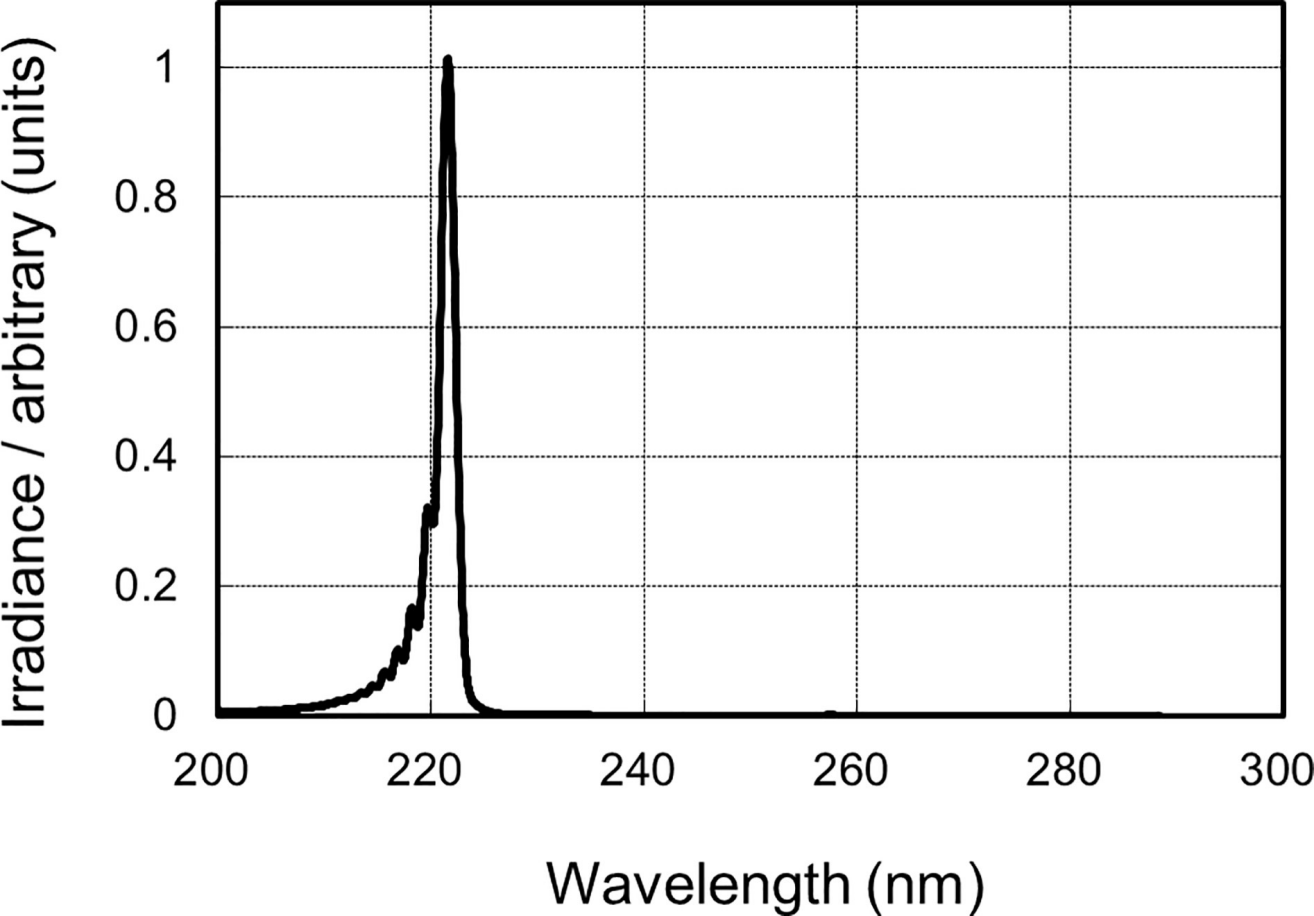
Spectra of the krypton-chloride excimer lamp. An optical filter which restricts spectra emitting light ranging from 200 to 230 nm, with the maximum output wavelength being 222 nm.

**Fig 3:**
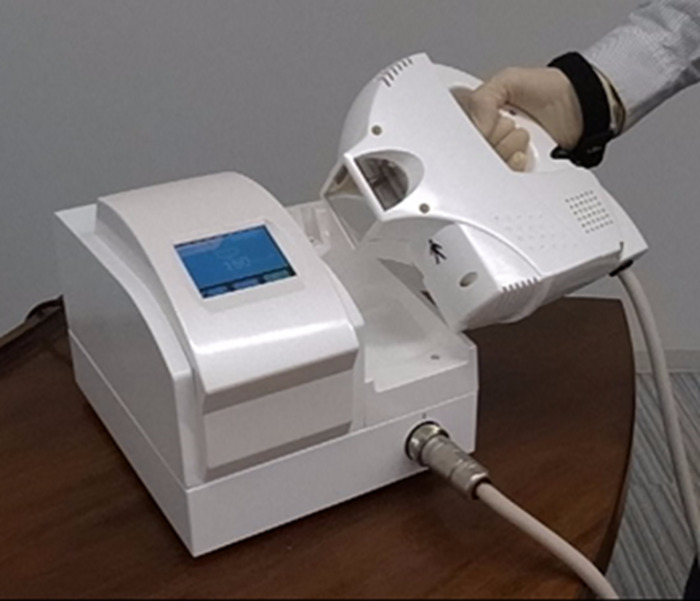
The 222-nm UVC irradiator. It has a handy irradiation port and can monitor the irradiation time. Abbreviation: UVC, ultraviolet radiation C.

A krypton-chloride (Kr-Cl) excimer lamp and an optical filter that restricts spectra emitting light ranging from 200 to 230 nm, with the maximum output wavelength being 222 nm, were used (Fig 2). The 222-nm-emitting SafeZoneUVC device (Ushio Inc. Tokyo, Japan), which was newly devised and prepared for this trial, is composed of a lamp, air-cooling fan, mirrors, and a custom band-pass filter (Fig 3).

The filter is used for blocking almost all wavelengths, excluding the dominant 222-nm emission wavelength. The irradiance emitted by 222-nm light was measured using an S-172/UIT250 accumulated UV meter (Ushio Inc.) and was observed to be 5.5 mW/cm^2^.

#### Method of UVC irradiation

The back area of the healthy subjects was irradiated at the portion between the third thoracic vertebra and the 12th thoracic vertebrae where no abnormality can be observed on the skin macroscopically. The currently proposed 222-nm UVC irradiation has never been used in humans; therefore, it is necessary to examine an appropriate irradiation dose that can demonstrate safety and a sufficient bactericidal effect. Considering that the minimum erythema dose of 254-nm UVC is 10 mJ/cm^2^, 500 mJ/cm^2^ was set as the maximum sufficient irradiation dose. In the event appearance of the erythema is observed at 500 mJ/cm^2^ or less in Step 1, the maximum irradiation dose at which erythema does not appear is set as the irradiation dose in Step 2 of this study. The irradiation sites of the doses were separately positioned from each other to avoid multiple irradiation being applied on the same area through this study.

#### Step 1–1: Erythema test with lower doses

UVC of 222 nm at 50, 100, and 200 mJ/cm^2^ was irradiated, and the presence or absence of erythema was examined 24 hours later. Compared to the non-irradiated sites, if barely discernable changes are observed at the irradiated site, the presence of erythema is ascertained. This was judged by two or more physicians, and the result was considered positive for erythema even when judged as such by one physician.

#### Step 1–2: Erythema test with higher doses

For cases in which no erythema was observed in Step 1–1, 222-nm UVC at 300, 400, and 500 mJ/cm^2^ was irradiated, and the presence or absence of erythema was investigated 24 hours later. The maximum dose that induced erythema in a participant was defined as the minimal erythema dose (MED), which is the smallest dose of radiation that could achieve faint but easily discernible erythema. Based on the lowest MED among all participants, the dose in Step 2 was determined, as shown in Table 2.

**Table 2:** MED in Step 1 and irradiation doses in Step 2.

MED in Step 1	Irradiation dose in Step 2
No MED (No erythema with 500 mJ/cm^2^)	500 mJ/cm^2^
500 mJ/cm^2^	400 mJ/cm^2^
400 mJ/cm^2^	300 mJ/cm^2^
300 mJ/cm^2^	200 mJ/cm^2^
200 mJ/cm^2^	100 mJ/cm^2^
100 mJ/cm^2^	50 mJ/cm^2^
50 mJ/cm^2^	Step 2 will be cancelled

Abbreviation: MED, minimal erythema dose.

#### Step 2

Irradiation with the maximum dose that induced no erythema in Step 1 was performed for all participants. Skin swab was collected before, and 5 and 30 minutes after irradiation and submitted for culture inspection. The skin tissue of the irradiated area was collected within 1 hour after irradiation with a biopsy under local anesthesia; in addition, DNA was extracted from the skin cells to measure generated cyclobutane pyrimidine dimer (CPD) by enzyme-linked immunosorbent assay (ELISA) as an indicator of DNA damage.

#### Skin-swab culture

The skin in the irradiated area was scraped with a swab (Pro-media st-25. Elmex Co., Ltd. Tokyo, Japan.), and the diluent of the swab was completely filtered through a 0.45-μm mixed cellulose ester membrane (EZ-Fit filtration unit, Merck, Darmstadt, Germany). The filtered membrane was placed on the surface of soybean casein digest agar (Nissui Pharmacy, Tokyo, Japan) in a plastic dish and was incubated at 37°C for 24 hours. The number of colonies formed in the dishes was counted macroscopically. Membrane filtration was applied since it was expected that the number of collected bacteria could be too small to be detected by swabbing of a narrow skin area.

#### Skin biopsy

Under local anesthesia, skin tissue was collected using a 3-mm diameter trepan for biopsy (BP-30F, Kai Industries Co., Ltd. Tokyo, Japan.) and quickly frozen.

*DNA collection and ELISA for CPD*. Collection of genomic DNA from the biopsy tissue was performed using the QIAamp Blood Kit (QIAGEN, Hilden, Germany. Cat. No. 51104) following the manufacturer’s instructions. The genomic DNA sample was sent to Cosmo Bio Inc., where the produced CPD amount measured with High Sensitivity CPD/Cyclobutane Pyrimidine Dimer ELISA kit Ver.2 (Cosmo Bio Inc., Tokyo, Japan. Cat No. NM-MA-K003) was compared among the irradiation group, the non-irradiation group, positive control, and negative control. The positive control was calf thymus DNA irradiated with 254-nm UVC of 1 mJ/cm^2^, whereas the negative control was non-irradiated calf thymus DNA.

### Data collection

The presence or absence of skin erythema 24 hours after the completion of 222-nm UVC irradiation of 500 mJ/cm^2^ or less was the primary outcome. The secondary outcomes included the presence or absence of a sterilization effect on the skin indigenous bacteria by the 222-nm UVC irradiation, presence or absence of DNA damage in skin cells after UV irradiation, and adverse events.

The irradiated regions were examined at least three months after the irradiation.

### Sample size estimates

This is the first study of its type to be conducted in human subjects, and there is no reference for the statistical estimation of the sample size. Therefore, the target number of cases was set at 20 to allow for a wide collection of indicators of safety and efficacy. As this study is an exploratory one for future research, we set a criterion such that this irradiation method is judged appropriately safe when the lower limit of the 95% confidence interval for erythema is higher than 75%. When erythema is not observed in all 20 participants, the lower limit of the 95% confidence interval is 83.2%.

### Statistical analysis

#### Analysis of safety

For the study of erythema, the proportion of erythema induced by 222-nm UVC irradiation and its 95% confidence interval were estimated. In the evaluation of DNA damage, the amount of CPDs generated in the 222-nm UVC irradiated- and non-irradiated areas of each participant was measured and statistically analyzed using a paired t-test. Other pairs of data were statistically analyzed using an unpaired t-test. The significance level of the hypothesis test was 5% two-sided.

#### Analysis of effectiveness

The frequency of bacterial detection by skin swabs was compared and examined using the Wilcoxon signed-rank test. The significance level of the hypothesis test was 5% two-sided.

## Results

### No erythema was induced by the 222-nm UVC irradiation of 500 mJ/cm^2^

In step 1 (erythema test), no erythema was observed in all 20 participants at all doses (50–500 mJ/cm^2^) of 222-nm UVC irradiation (0%, 95% confidence interval [0% to 16.8%]). Based on this result, 500 mJ/cm^2^ was used as the dose for step 2.

### 222-nm UVC irradiation has a bactericidal effect on the human skin

**Fig 4:**
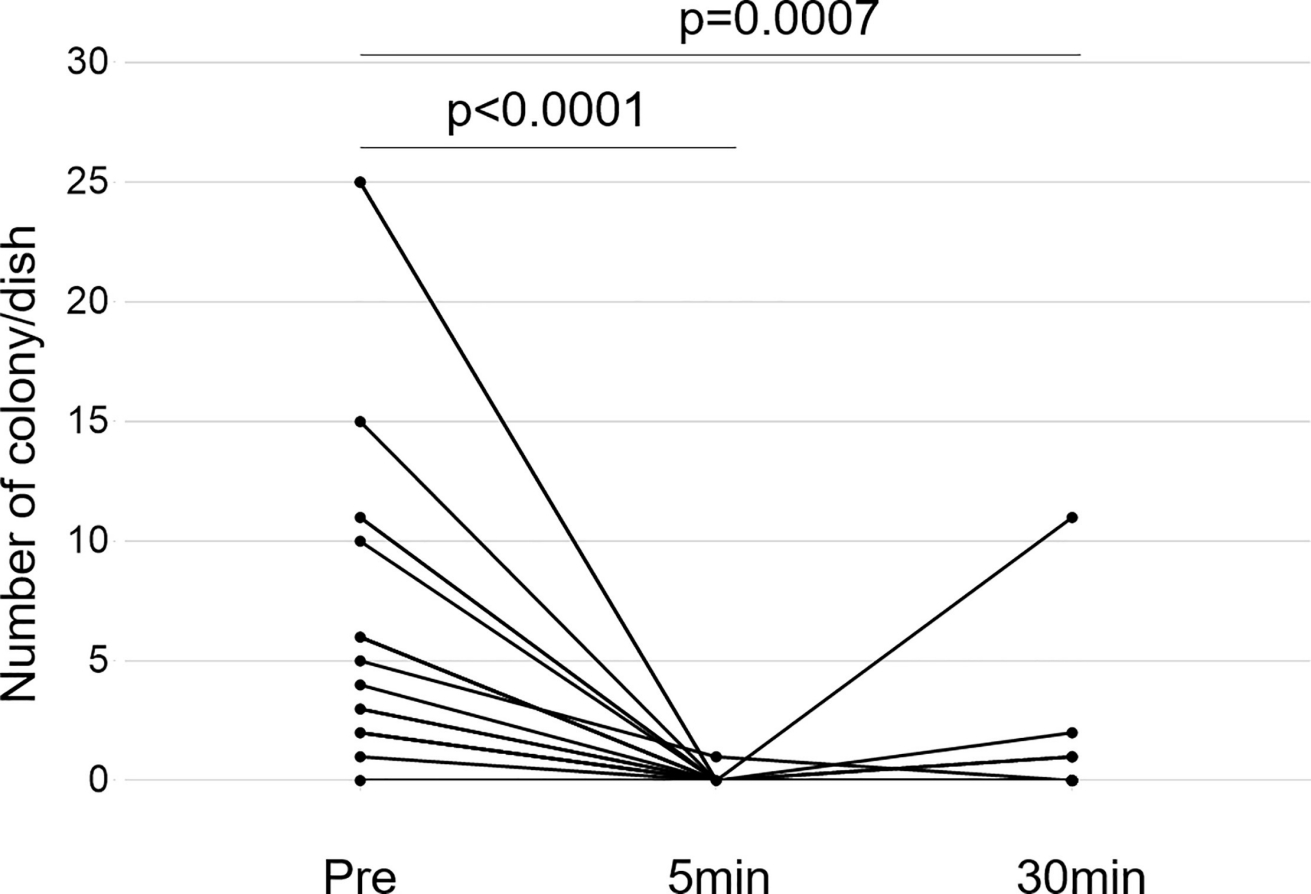
Bacterial colony numbers in cultured skin swab suspension. The mean number and standard deviation of colonies formed pre-irradiation, 5 minutes after irradiation, and 30 minutes after irradiation were 7.21±7.48, 0.05±0.23, and 0.79±2.53, respectively. Each data is shown as a black spot, and individual pair of data are connected with lines. The colony number was significantly decreased by 222-nm UVC irradiation at 5 and 30 minutes after the irradiation than at the pre-irradiation. No significant difference was observed in the colony number between 5- and 30-minute post-irradiation skin swab cultures. Abbreviation: UVC, ultraviolet radiation C.

In all 20 sets of samples, one set was accidentally discarded before the colony number was counted. Therefore, the assessment was performed in the samples of the 19 participants. The colony number on the membrane was significantly lower in both 5- and 30-minute post-irradiation skin-swab cultures than in the pre-irradiation skin-swab culture (Fig 4).

### CPD amount by UVC irradiation was slightly higher than that without irradiation

**Fig 5:**
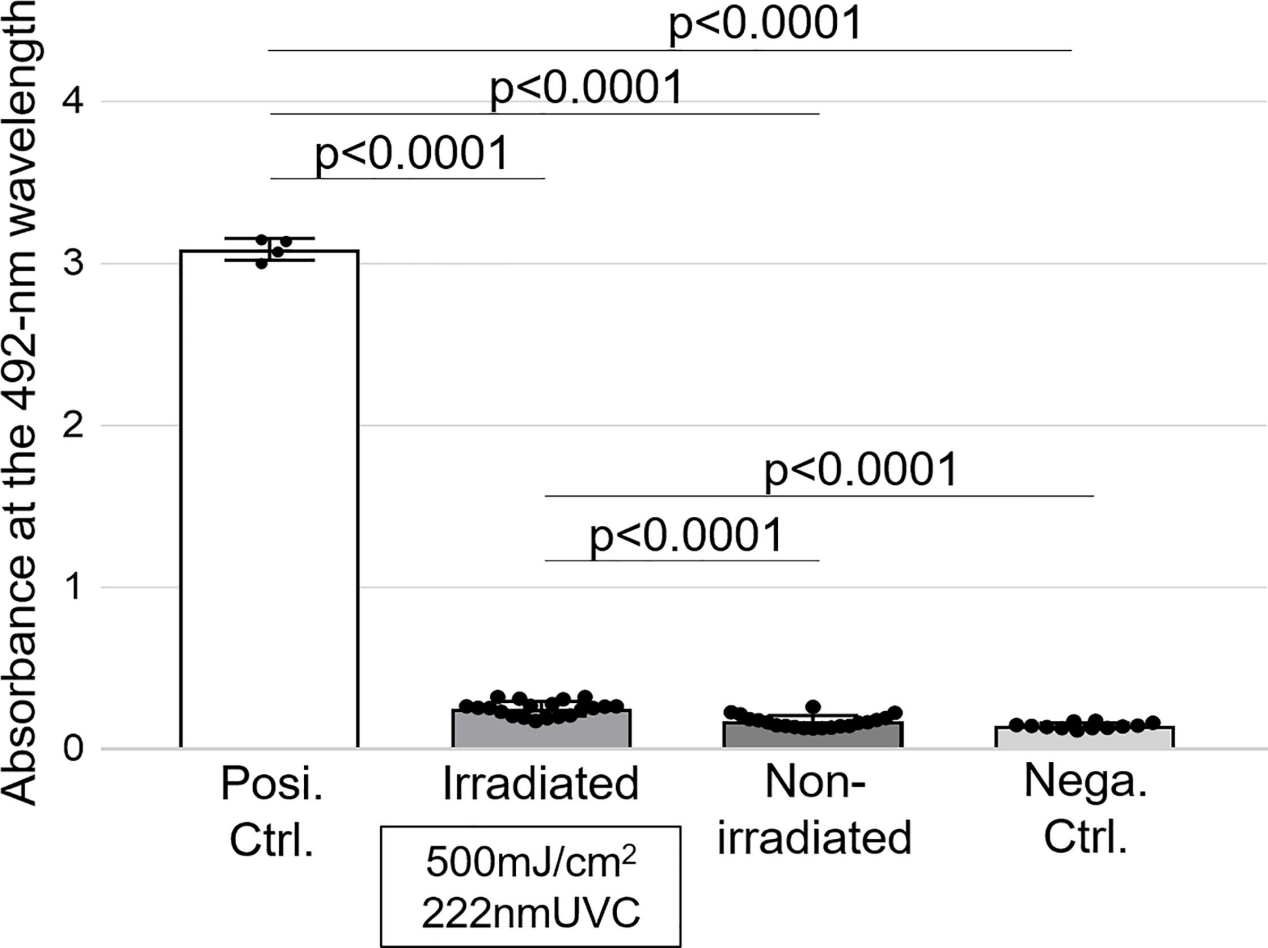
CPD amount evaluated by ELISA. The absorbance at a wavelength of 492-nm is proportional to the CPD amounts. The mean absorbance and standard deviation of positive control, irradiated samples, non-irradiated samples, and negative control was 3.09±0.07, 0.25±0.05, 0.17±0.04, and 0.14±0.02, respectively. Each data is shown as a black spot. It is shown that the absorbance of the positive standard was significantly greater than that of the other three samples, and that of the irradiated region was significantly greater than that of the non-irradiated region and negative control. No significant difference was observed between the non-irradiated region and negative control. Abbreviation: ELISA, enzyme-linked immunosorbent assay.

The amount of CPD produced was evaluated and compared among the irradiated region, non-irradiated region, negative control, and positive control. Compared with the positive control, CPD amounts in the other three groups were significantly low. The amount of CPD produced in the irradiated region was slightly but statistically significantly higher than that in the non-irradiated region and negative control (Fig 5).

### Three-month macroscopic follow-up after irradiation

Follow-up assessment at 3 months post-irradiation demonstrated that no skin symptom, including erythema was observed in all participants. Similarly, no adverse event was noted.

## Discussion

To achieve the clinical application of the 222-nm UVC irradiation on the surgical wound for SSI prevention, a study examining the method’s safety and effect is required. This study was conducted using normal human skin in the first step, before applying the method on the surgical wound for its clinical application. To our knowledge, this is the first study investigating the safety and bactericidal effect of 222-nm UVC in human subjects. In this study, the erythema test demonstrated that 222-nm UVC irradiation at 500 mJ/cm^2^ or less induced no erythema, indicating that this irradiation dose is lesser than the dose that could induce sunburn. The skin-swab test was performed at 5 and 30 minutes after the irradiation, and the swab culture during these time points were compared with the skin swab of the non-irradiated area. Both time points showed significantly lower colony numbers than the non-irradiated area, indicating that 222-nm UVC irradiation at 500 mJ/cm^2^ has a bactericidal effect on the human skin, which lasts for at least 30 minutes after the irradiation. Combining the results of erythema and skin-swab tests indicated that a sunburn-free dose of 222-nm UVC can sterilize the skin safely.

It has been long known that UVC has a bactericidal effect. In fact, UVC has been utilized as a germicidal lamp. The 254-nm UVC irradiators are clinically used in the United States and Canada for the treatment of pressure ulcer with infection. Concurrently, since UVC is cytotoxic, it is feared that malignant tumors, such as melanoma and eye disorders (e.g. keratitis) may develop [14,16]. Based on this background, we considered 222-nm UVC a safe wavelength for irradiating the human skin. This implies that since more doses of 222-nm UVC can be irradiated safely, the 222-nm UVC irradiation is supposedly a more powerful irradiation.

The safety of 222-nm UVC was investigated in animal experiments conducted by other institutions. Buonanno et al. performed irradiation of 254-nm and 222-nm UVC at 157 mJ/cm^2^ on hairless mice and evaluated generated CPDs and 6–4 photoproducts as markers of DNA damage. The study result revealed that the generation of CPDs and 6–4 photoproducts was exclusively observed after irradiation of 254-nm UVC, with DNA damage being confirmed [17]. Another previous study similarly reported that even chronic irradiation of 222-nm UVC at 450 mJ/cm^2^/day for a total of 10 days induced no DNA damage in mice [18].

These data indicate the safety of 222-nm UVC irradiation on the area with stratum corneum. However, to apply this method in the prevention of SSI, the irradiation should be tested on an area without stratum corneum, such as the general surgical sites. Human cells are as large as 5–25 μm, whereas the size of bacteria is 1 μm or less. It has been previously reported that 157 mJ/cm^2^ of 222-nm UVC does not reach the human nucleus, thereby inducing no DNA damage [17,19,20]. Therefore, irradiation of 222-nm UVC on a wound without stratum corneum is theoretically considered safe.

Irradiation on wound without stratum corneum was studied by Narita et al. The photo-genotoxicity of the cell nucleus using CPD as a marker following 500 mJ/cm^2^ of 222-nm UVC on the normal skin and area without epidermis was investigated and compared with that of a case with 254-nm UVC irradiation [21]. The experimental results revealed CPD was not detected after 222-nm UVC irradiation at 500 mJ/cm^2^ on the normal skin and that CPD was detected in 60% of keratinocytes after 254-nm UVC irradiation. Moreover, CPD was detected in 80% of fibroblasts 1 hour after 254-nm UVC irradiation; on the contrary, CPD was not detected after irradiating the wounds without epidermis with 500 mJ/cm^2^ of 222-nm UVC.

Although previous studies involving animals reported that 222-nm UVC irradiation induced no CPD, ELISA in this study showed slight but statistically significant increase in CPDs in the irradiated region than in the non-irradiated region. This discrepancy in findings could be due to differences in the species used as subjects and in the doses of 222-nm UVC irradiation. Moreover, the main cause of discrepancy was supposedly the different methods of CPD assessment used in this study and previous ones. Previous studies performed a histological assessment to evaluate CPD, whereas ELISA was used in this study. It is speculated that ELISA is more sensitive than the histological assessment for detecting CPDs generation. CPD generation in the human skin cells is estimated to occur daily, especially when staying outdoors for 20 minutes on a sunny day, as previously reported [22]; therefore, the generation of CPDs does not necessarily indicate that the activity is harmful provided that it is within the amount of repairability. In this study, CPD was slightly detected in the human ELISA. However, considering the property of 222-nm UVC on human cells, CPD formation is theoretically supposed to occur in the upper layer of the epidermis [13,17,19,20]. In this study, ELISA revealed that the CPD value obtained by subtracting the CPD value of the negative control from that after the 222-nm UVC irradiation at 500 mJ/cm^2^ is only 3.5% of the CPD value of the positive control. A previous study utilizing histochemistry data, rather than ELISA data, reported that irradiation of 254-nm UVC at 150 mJ/cm^2^ on the artificial skin and at 157 mJ/cm^2^ on the mouse skin induced CPD production in 50% and 52.3% of keratinocytes, respectively [17]. Moreover, another study reported CPD-expressing cells in the keratinocytes as approximately 60% after 254-nm UVC irradiation at 150 mJ/cm^2^ on the mouse skin [21]. Compared to the detected CPD amount after 254-nm UVC irradiation in these studies, that generated by 222-nm UVC irradiation in this study was remarkably low despite a high irradiation dose even assuming the positive control of this report produces CPD at 100% in keratinocytes. In this study, although it was impossible to perform histological assessment with human samples, we have obtained data from histological investigations on CPD localization in another series of study [23]. To achieve the clinical application of this method for SSI prevention, an animal study examining the safety of 222-nm UVC irradiation on a surgical wound model is warranted.

## Conclusions

Irradiation of 222-nm UVC 500 mJ/cm^2^ is safe and has a bactericidal effect on the human skin. It is expected to be a new modality for preventing SSI in clinical settings.

## Supporting information

S1 Checklist (PDF)

S1 File (PDF)

S2 File (DOCX)

S3 File (PDF)

S4 File (DOCX)
