# Supplementary material for: Exploratory clinical trial on the safety and bactericidal effect of 222-nm ultraviolet C irradiation in healthy humans
Source: PLoS One. 2020 Aug 12;15(8):e0235948. doi: 10.1371/journal.pone.0235948 (PMC7423062; doi:10.1371/journal.pone.0235948)
Supplement: S2 File — (DOCX) [file pone.0235948.s003.docx]

**Exploratory clinical trials on the safety and bactericidal effect of 222 nm UVC irradiation on healthy humans.**

Study protocol

Ver. 1.0. May 22. 2017.

| Research contact person |
| --- |
| Name：Takahiro Niikura  Institute：Department of Orthopaedic Surgery, Kobe University Graduate School of Medicine.  TEL：+81-78-382-5985  FAX：+81-78-351-6944  E-mail：[tniikura@med.kobe-u.ac.jp](mailto:tniikura@med.kobe-u.ac.jp) |

| Public contact |
| --- |
| Institute: Department of Orthopaedic Surgery, Kobe University Graduate School of Medicine.  Address：7-5-2, Kusunoki-cho, Chuo-ku, Kobe, JAPAN  TEL：+81-78-382-5985  FAX：+81-78-351-6944  E-mail：[tniikura@med.kobe-u.ac.jp](mailto:tniikura@med.kobe-u.ac.jp) |

Contents

[**1．**](#_Toc431378516)[**Background** 3](#_Toc431378517)

[**2．Objective and necessity of the trial**](#_Toc431378517) 5

[**3．****Equipment**](#_Toc431378518) 5

[**4．Subjects**](#_Toc431378519) 6

[**5．M****ethod to obtain informed consent**](#_Toc431378520) 7

[**6．Trial method**](#_Toc431378521) 8

[**7．Endpoints**](#_Toc431378522) 9

[**8．Survey items** 10](#_Toc431378523)

[**9．Discontinuation criteria**](#_Toc431378524) 12

[**10．Handling of adverse events at onset** 13](#_Toc431378525)

[**11．Completion, termination, and suspension of the trial** 14](#_Toc431378526)

[**12．Study implementation period** 1](#_Toc431378527)5

[**13．Subject analysis (sub-group for effectiveness evaluation) and method of statistical analysis (primary/secondary analysis)** 15](#_Toc431378528)

[**14．Quality assurance of the trial** 17](#_Toc431378529)

[**15．****Consideration of human rights and safety/disadvantages to the subjects** 17](#_Toc431378530)

[**16．Expenses for the subjects** 1](#_Toc431378531)8

[**17．Correspondence to** **ethical guidelines and Declaration of Helsinki** 1](#_Toc431378532)8

[**18．Retention of recorded documents** 1](#_Toc431378533)8

[**19．Registration of research program** 18](#_Toc431378534)

[**20．Research organization** 18](#_Toc431378535)

[**21．Disclosure of research funding sources and COI status of researchers** 19](#_Toc431378536)

[**22．Change of study protocol** 20](#_Toc431378537)

[**23．****Publication of research results**](#_Toc431378538) 20

[**24．References**](#_Toc431378539) 20

# **1．Background**

Surgical treatment accounts for a large proportion of modern medical treatments and many patients require it. Various surgical treatments are carried outin several departments regardless of domestic and overseas, but there is a risk of various complications, and one of the most serious is perioperative infection. Once infection is established, it is difficult to treat., and both patients and medical professionals will be annoyed with great burden. Despite the fact that some measures have been taken to prevent perioperative infections, such as the administration of antibiotics based on guidelines in recent years, perioperative infections have frequently occurred in daily practice and the establishment of a solid strategy to prevent them is still required.

Perioperative infections may be caused by environmental factors, patient factors, microbial factors, etc. ^1^, but bacteria are a major causative agent of perioperative infections. These include endogenous factors such as resident bacteria on the skin and the nasal mucosa of patients as well as exogenous bacteria, such as airborne bacteria. Although no consensus has been reached as to which of these is more important, there are many reports highlighting the involvement of endogenous bacterial infections^2,3,4^. Even in a surgical field that has been sufficiently disinfected before surgery, it is necessary to continuously try to prevent the contamination of surgical wounds caused by the proliferation of skin resident bacteria during surgery.

However, it has long been known that ultraviolet (UV) irradiation has a bactericidal effect. Bacterial growth is stopped, leading to death by the dimerization of adjacent bases of bacterial DNA and RNA. In particular, UV type C (UVC), having a short wavelength of 200 to 280 nm, has a high bactericidal effect due to its high absorption coefficient by DNA, and is used as a bactericidal lamp. In fact, in the United States and Canada, a device that emits UVC of 254 nm has been approved and is used clinically as a treatment device for wounds with infection. However, UVC irradiation is cytotoxic and there are concerns about the risk of developing malignant tumors, so a safer method is desired.

When 254 nm UVC irradiates the skin, it passes through the stratum corneum and reaches the epidermis, thus affecting the epidermal cells. However, 222 nm UVC has a high protein absorption coefficient and only penetrates down to the stratum corneum, which is the outermost layer of the epidermis, so it is considered to be a very safe UVC that does not affect skin cells. Even if irradiating wounds without stratum corneum, human cells are as large as 5-25 μm compared to bacteria less than 1 μm, the 222 nm UVC does not reach the human cell nucleus, so is considered safe because it will not cause damage to the DNA. For this reason, a larger amount of irradiation of 222 nm UVC is possible.


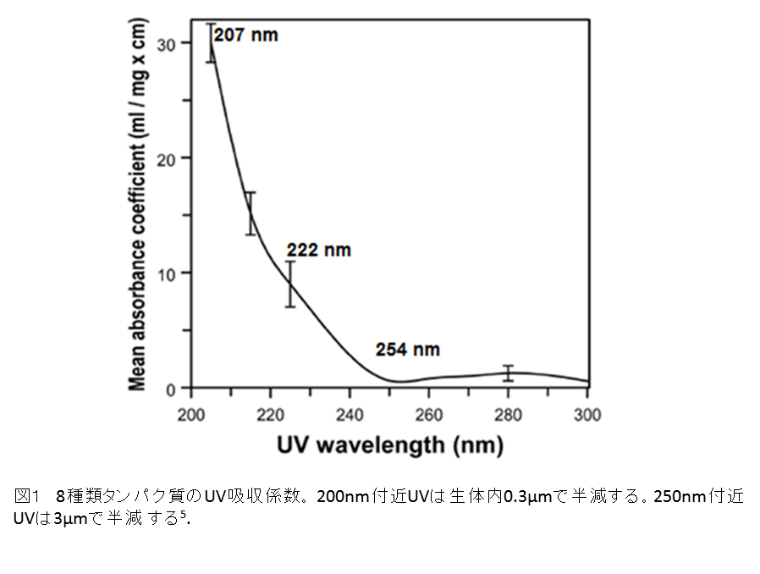


Figure 1: UV absorption coefficient of eight kinds of proteins. UV near 200 nm is halved at 0.3 μm *in vivo*. UV near 250 nm is halved at 3 μm.^5^

However, the sterilization ability of 222 nm and 254 nm UVC at the same irradiation dose is equivalent, as shown in Figure 2.


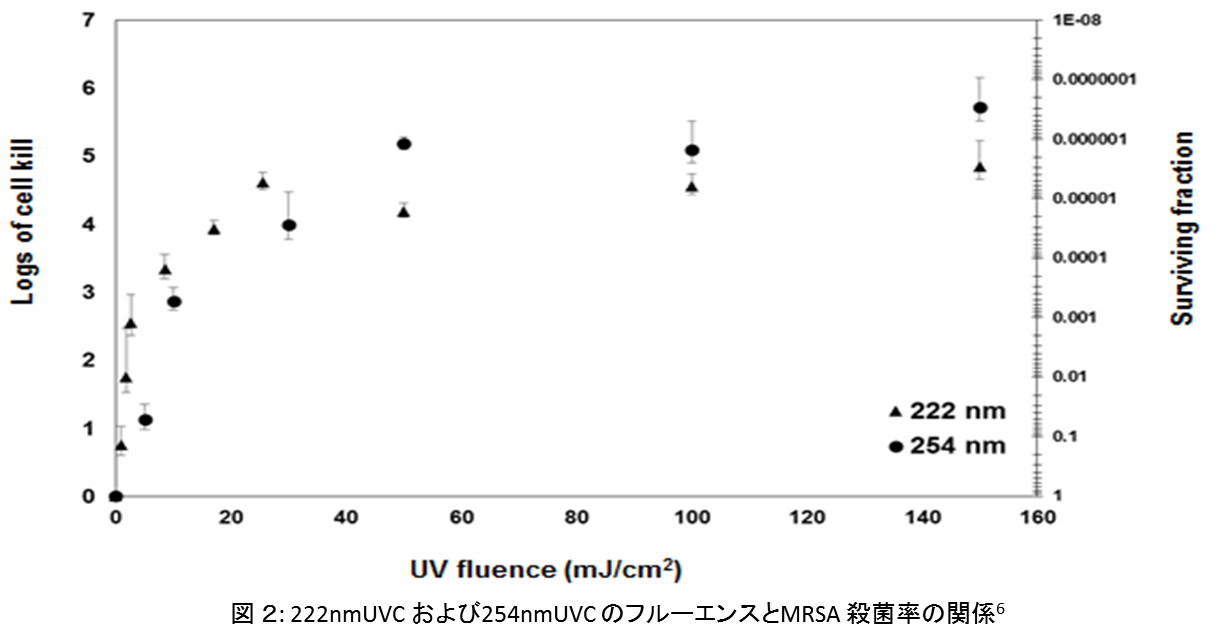


Figure 2: Relationship between MRSA surviving rate and UV dose of 222 nm and 254 nm.^6^

Based on these data, 222 nm UVC is expected to have better bactericidal properties than 254 nm UVC.

Animal experiments regarding safety of UV irradiation were carried out by Buonanno M et al. from Columbia University, and Narita et al. from Hirosaki University. Buonanno M et al. irradiated hairless mice using both 254 nm and 222 nm UVC at a dose of 164 mJ/cm^2^. They measured the formation of cyclobutane pyrimidine dimer (CPD) and 6-4photoproducts (6-4PP) as UV damage markers for cell nuclei (DNA); CPD and 6-4PP production was observed, suggesting that DNA damage was confirmed.^6^

Narita et al. irradiated normal skin and non-epidermal wounds in mice using 222 nm UVC at a dose of 500 mJ/cm^2^. They examined cell nuclei using CPD as a marker and compared it with the effects of 254 nm UVC irradiation.^7^。

The experimental results were as follows.

1. CPD was not detected in normal skin after irradiation with 222 nm UVC at a dose of 500 mJ/cm^2^. With 254 nm UVC irradiation, CPD was detected in 60% of keratinocytes. (Figure 3)
2. CPD was not detected in wounds after irradiation with 222 nm UVC at a dose of 500 mJ/cm^2^. After irradiation with 254 nm UVC, CPD was detected in 80% of fibroblasts after 1 hour. (Figure 4)

Regarding irradiation on humans, the human proof of concept (POC) study for bacterial management of pressure ulcers has started at the National University of Singapore.^8^ The device used is the same device developed by Ushio Inc. The amount of irradiation at one time is 500 mJ/cm^2^, and irradiation is performed once every two days for two weeks. Currently, two patients have completed irradiation, and a decreased amount of bacteria was observed after irradiation. No erythema was observed in the normal skin around the wound after irradiation.


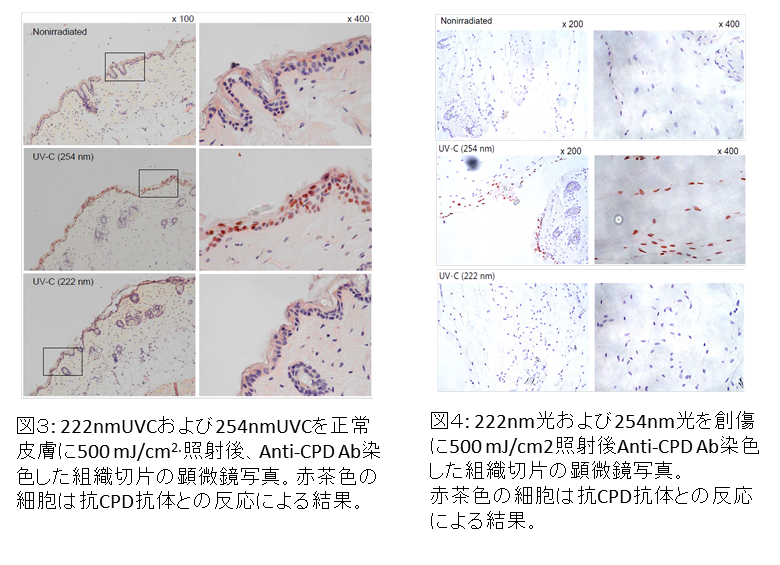


Figure 4: Photomicrographs of tissue sections of wounds stained with anti-CPD Ab after irradiation with 222 nm and 254 nm UVC at a dose of 500 mJ/cm^2^. Red-brown cells are the result of the reaction with anti-CPD antibody.

Figure 3: Photomicrographs of tissue sections of normal skin stained with anti-CPD Ab after irradiation with 222 nm and 254 nm UVC at a dose of 500 mJ/cm^2^. Red-brown cells are the result of the reaction with anti-CPD antibody.

Woods JA et al. reported irradiation experiments on normal human skin using a 222 nm KrCl excimer lamp^9^. In this report, they show that CPD generation in the skin tissue in the irradiated range in some subjects, but they conclude that it was caused by the presence of UVC with a wavelength longer than 222 nm.

We hypothesized that 222 nm UVC irradiation may dramatically improve the perioperative infection rate and thus initiated a study to clinically apply a device (a UVC irradiator developed by Ushio Inc.) that irradiates the surgical site with a 222 nm. This device is not like the irradiator reported by Woods et al., mentioned above, but is like the equipment used at the National University of Singapore that contains a filter to remove UVC wavelengths other than 222 nm, thus having a more specific effect.

# **2．Objective and necessity of the trial**

To investigate the safety of 222 nm UV irradiation using a UVC irradiator to examine its bactericidal action on the skin of healthy human volunteers.

# **3.** **Equipment**

A 222 nm UVC irradiator. Details are described in another document.

1）Equipment information:

A 222 nm UVC irradiator from Ushio Inc.

2）Expected adverse reactions and expected malfunctions of medical devices:

Theoretically, adverse reactions are unlikely to occur, but there is no evidence that denies the appearance of human skin damage and genetic abnormalities in skin cells caused by 222 nm UV irradiation using a UVC irradiator.

# **4．Subjects**

Those who meet all of the following inclusion criteria and do not fall under any of the following exclusion criteria are the subjects of this study.

1. Inclusion criteria：

1）Those who are between 20 and 80 years old at the time of obtaining consent.

2）Gender: both female and male.

3）Those who provided documented consent by their voluntary intention to participate in this trial.

2）Exclusion criteria：

1) Sensitivity to drugs, such as allergies.

2) Abnormality at the site of UVC irradiation on the back, such as dermatitis or trauma.

3) Regular use of topical medication or patches at the site of UVC irradiation on the back.

4) Tattoos at the site of UVC irradiation on the back.

5) Pregnancy, the possibility of pregnancy or breast-feeding.

6) Participation in other clinical trials within four months.

7) Rejection as inappropriate for participation by the physician in charge of the clinical trial.

Rationale:

2) Set to ensure the safety of subjects.

4) Set because there is a possibility of hindering the evaluation of changes by UV irradiation.

5) Set because the safety of the equipment for pregnant women and fetuses has not been confirmed.

6) Set in consideration of ethical issues for subjects participating in clinical trials.

7) Set to leave room for the investigator to decide whether or not the subject can participate in this study, taking into account all other factors than those mentioned above and ensuring the safety of the subjects.

3）Target sample size:

20

4）Procedure of enrolling subjects:

To ensure safety, UV irradiation will be performed on two subjects and they will be checked for the presence or absence of erythema 24 hours after irradiation. If there is no situation where erythema appears with all irradiation dosesthe number of patients enrolled in stages will be increased. Four subjects first, followed by six and eight subjects.

# **5．Method to obtain informed consent**

Prior to the start of the study, the principal investigator or the sub-investigators will fully explain the following to the candidates both in writing and verbally. For the explanation, the principal investigator or the sub-investigators will use the consent explanation document approved by the ethics review committee. Candidates should be given the opportunity to ask questions and have sufficient time to decide whether or not to agree, and after confirming that they have fully understood the contents of the study, the principal investigator or the sub-investigators will obtain their consent by their own free will. When consent is obtained from the subject candidate, the signature or name seal on the consent document from the subject candidate and the date of consent will be filled in. The explainer (the principal investigator or the sub-investigators) will also sign or seal the consent document and will fill in the date of explanation. The originals of the consent document with the signature or seal of the subject and the explainer and the date entered will be kept with the study secretariat and a copy will be given to the subject and the surrogate.

1. Introduction（Background research of the study）
2. Purpose of the clinical trial
3. Method of the clinical trial
4. Number of participants in the trial
5. Expected effects
6. Expected side effects and risks
7. In case your health is damaged during this trial
8. Disclosure of information such as genetic analysis and expected benefits

and disadvantages

1. Expenses associated with this trial
2. Candidates will not suffer any disadvantage, even if they disagree
3. Withdrawal after consent
4. Retention, usage, and retention period of samples
5. Disclosure of trial protocol
6. Privacy protection
7. Viewing personal information
8. Intellectual property rights attribution
9. Funding sources and conflicts of interest regarding the trial
10. What the candidate can keep
11. Study secretariat
12. Research organization and the principal investigator

# **6．Trial method**

1）Study design

Exploratory clinical trial

2）Outline of the trial

This study is an exploratory trial to investigate the safety and efficacy of 222 nm UVC irradiation on healthy human volunteers using the UVC irradiator from Ushio Inc.

Outline of the study schedule is shown in Figure 5.

Trial is started after obtaining consent.

＜Step 1＞

Determine irradiation dose in 2^nd^ step

UVC irradiation

|  | Immediately before irradiation | 24 hours later |
| --- | --- | --- |
| Erythema check | 〇 | 〇 |

＜Step 2＞

UVC irradiation

Measurement of CPD in genomic DNA collected seven days after irradiation

|  | Immediately before irradiation | 5 minutes later | 30 minutes later | 1 hour later |
| --- | --- | --- | --- | --- |
| Skin swab | 〇 | 〇 | 〇 |  |
| Biopsy |  | 〇 | | |

Figure 5. Outline of the study

3）Study implementation period

Target subject number：20

Enrollment period：11 months. Follow-up period：Approximately 1 month after start of the trial (until result of CPD test is revealed.)

Total study implementation period: 12 months.

4）Procedures of UV irradiation

The back area of healthy subjects will be irradiated at the portion between the third thoracic vertebra and the 12th thoracic vertebrae where no abnormality of the skin can be found macroscopically. The purpose of this study is to investigate the safety of 222 nm UVC irradiation using the UVC irradiator from Ushio Inc. It is theoretically assumed that erythema and DNA damage to skin cells will not occur; therefore irradiation of a sufficient dose is necessary. Considering that the minimum dose of 254 nm UVC causing erythema is 10 mJ/cm^2^, a dose of 500 mJ/cm^2^ is set as the maximum sufficient irradiation dose.

If the appearance of erythema is observed at 500 mJ/cm^2^ or less, the maximum irradiation dose at which erythema does not appear will be set as the irradiation dose in a following study.

5）Rules for concomitant drugs (therapy)

1. Concomitant drugs (therapy): There are no provisions regarding concomitant drugs (therapy).
2. Prohibited drugs (therapy): The use of UV irradiation equipment other than that used in this study is prohibited.
3. Drugs (therapy) requiring attention in combination: There are no provisions regarding drugs (therapy) requiring attention in combination.

6）Retention of samples etc.

The principal investigator and the sub-investigators will retain the samples (biological sample, specimens), equipment, etc. related to the conduct of the trial for five years from the date when the final publication of the results of this study is reported, and then will dispose of them in an unidentifiable state.

The samples and data obtained from the subjects in this study may be used in another study in the future. In that case, that study will be conducted after a full explanation is given to the research subjects.

・Institution providing samples and data：Department of Orthopaedic Surgery, Kobe University Hospital

・Institution samples and data will be provided to：Ushio Inc., Cosmo Bio Co., Ltd.

# **7．Endpoints**

1）Primary endpoint
・Presence or absence of skin erythema at 24 hours after UV irradiation with 500 mJ/cm^2^ 222 nm UVC or less.

2）Secondary endpoints

1. Bactericidal effect of skin indigenous bacteria by UV irradiation.

2. DNA damage in skin cells after UV irradiation.

3. Adverse events.

# **8．Survey items**

（1）Observation and inspection items to be collected during the test

1) At the time of informed consent

- The identification code of the subject will be confirmed, gender, date of birth, height, weight, complications, and medical history will be checked, and study eligibility will be assessed.

＜Step 1＞

2) Before irradiation

- Specify the irradiation range of six places (E1, E2, E3, E4, E5, E6) on the back of the subject. Since the irradiation dose does not depend on the irradiated area, it is not necessary to set a strict size of the area. Specify the range (N) on the subject's back where UV is not irradiated. (Figure 6)

3) UV Irradiation

- Doses of 50, 100, and 200 mJ/cm^2^ 222 nm UVC will be irradiated onto E1, E2, and E3, respectively. The amount of irradiation can be measured with the irradiation equipment itself. The time required for irradiation is approximately 10 seconds at 50 mJ/cm^2^, approximately 20 seconds at 100 mJ/cm^2^, and approximately 40 seconds at 200 mJ/cm^2^. (Figure 6）

Figure 6. Irradiated area and irradiation doses ①

|  | UVC (+) 50 mJ/cm^2^ | UVC (+) 100 mJ/cm^2^ | UVC (+) 200 mJ/cm^2^ | UVC (-) |
| --- | --- | --- | --- | --- |
| Erythema | **E1** | **E2** | **E3** | **N** |

4) 24 hours after irradiation

- The presence or absence of erythema in E1, E2, and E3 will be evaluated and compared with N. A photograph of the non-irradiated and irradiated area will be retained. If no erythema is observed after 24 hours, proceed to step 5 (next). If erythema is observed, the minimum dose that causes erythema will be taken as the minimum erythema dose (MED), and the UVC dose in step 2 will be determined as in Figure 8.

5) UV irradiation

- Doses of 300, 400, and 500 mJ/cm^2^ of 222 nm UVC will be irradiated onto E4, E5, and E6, respectively. The time required for irradiation is approximately 60 seconds at 300 mJ/cm^2^, approximately 80 seconds at 400 mJ/cm^2^, and approximately 100 seconds at 500 mJ/cm^2^. (Figure 7)

Figure 7. Irradiated area and irradiation doses ②

|  | UVC (+) 300 mJ/cm^2^ | UVC (+) 400 mJ/cm^2^ | UVC (+) 500 mJ/cm^2^ | UVC (-) |
| --- | --- | --- | --- | --- |
| Erythema | **E4** | **E5** | **E6** | **N** |

6) 24 hours after the irradiation

- The presence or absence of erythema in E4, E5, and E6 will be evaluated and compared with N. A photograph of the non-irradiated and irradiated area will be retained. If erythema is observed, the MED will be set and the UVC dose in step 2 will be determined as in Figure 8. After determination of the MED for all 20 cases, Step 2 will be performed.

7) Determination of the irradiation dose in step 2

- Based on the lowest MED in all subjects, the irradiation dose in step 2 will be determined as in Figure 8.

| The lowest MED in all subjects | Irradiation dose in step 2 |
| --- | --- |
| No MED (No erythema with 500 mJ/cm^2^) | 500 mJ/cm^2^ |
| 500 mJ/cm^2^ | 400 mJ/cm^2^ |
| 400 mJ/cm^2^ | 300 mJ/cm^2^ |
| 300 mJ/cm^2^ | 200 mJ/cm^2^ |
| 200 mJ/cm^2^ | 100 mJ/cm^2^ |
| 100 mJ/cm^2^ | 50 mJ/cm^2^ |
| 50 mJ/cm^2^ | Discontinuation without step 2 |

Figure 8. MED in Step 1 and irradiation doses in Step 2

＜Step 2＞

8) UV irradiation

- Specify the irradiation range (40 x 40 mm) at three locations (S5, S30, B) on the back of the subject and irradiate with 222 nm UVC using the irradiation dose determined in 7. (Figure 9)
- Swab the surface of N0 with a swab (Elmex, Pro-media st-25) and submit it for culture testing.

40 mm

40 mm

NB

S5

S30

B

10 mm diameter

N5

N30

40 mm

40 mm

|  | UVC (+) | UVC (-) |
| --- | --- | --- |
| Swab：Pre-irradiation |  | **N0** |
| Swab：5 minutes after irradiation | **S5** | **N5** |
| Swab：30 minutes after irradiation | **S30** | **N30** |
| Biopsy：1 hour after irradiation | **B** | **NB** |

Figure 9．Irradiated area and irradiation doses➂

9) 5 minutes after irradiation

- Swab the surface of S5 and N5 with a swab and submit it for culture testing.

10) 30 minutes after irradiation

- Swab the surface of S30 and N30 with a swab and submit it for culture testing.

11) 1 hour after irradiation

- B and NB are locally anesthetized, skin tissue will be collected using a 3 mm diameter biopsy trepan (KAI Medical Co., Ltd., BP-30F) and immediately fixed in formalin. Procced to step 12.

12) By seven days after biopsy

- Collect genomic DNA from biopsy tissue with QIAamp Blood Kit (QIAGEN, Cat. No. 51104 or 51106). The concentration of genomic DNA will be measured.
- The genomic DNA sample will be sent to Cosmo Bio Inc. and CPD (cyclobutane pyrimidine dimer), which is an index of DNA damage caused by UV irradiation, will be measured. The amounts of CPD produced will be compared between the irradiated group and the non-irradiated group, and if the amount of CPD produced is increased with irradiation, with statistical significance, it will be determined that there is DNA damage.

# **9．Discontinuation criteria**

When continuation of the study is judged to be impossible for any of the following reasons, the principal investigator or sub-investigator will discontinue the subject from the study and specify the date/time of discontinuation/dropout, reason for discontinuation/dropout and clinical course, in the medical record and case report form (CRF). In addition, at the time of the discontinuation/dropout, the principal investigator or sub-investigator will perform the necessary tests and assess efficacy and safety.

① When the subject requests to withdraw from the study or withdraws consent

② When the subject is found not to meet the eligibility criteria after enrollment

③ When a concurrent disease worsens and further continuation with the study is difficult

④ When an adverse event occurs and further continuation with the study is difficult

⑤ When pregnancy is found

⑥ When the study itself is discontinued

⑦ When discontinuation from the study is appropriate for other reasons in the opinion of the principal investigator or sub-investigator

# **10．Handling of adverse events at onset**

When an adverse event is noted, the principal investigator or sub-investigator will immediately perform the appropriate treatment and will record the adverse event in the CRF without discrepancies. In addition, when the UV irradiation is discontinued or if the adverse event requires treatment, this should be explained to the subject. The principal investigator and the sub-investigators will be responsible for following up adverse events in all subjects, regardless of their relevance to UV irradiation, until the symptoms disappear, or otherwise (permanent/irreversible adverse events, etc.), and until sufficient explanation is given. Adverse event terms, severity, seriousness, causal relationships with UV irradiation (not related or related) should be recorded.

At the final examination (including adverse events that occurred after UV irradiation and the end of observation), treatment and outcome related to UV irradiation will be recorded. If DNA damage is observed, re-irradiate with the same dose, perform biopsy 48 hours after irradiation, and measure CPD. If there is a significant difference in the amount of CPD produced between the irradiated group and the non-irradiated group, observe the irradiated site for abnormalities for 3 months after irradiation.

The follow-up period for adverse events is the period until the subject recovers from the adverse events or until the investigator and the sub-investigators decide that further follow-up is unnecessary.

・Report of serious adverse events

A serious adverse event is defined as any event that:

(1) results in death,

(2) is life-threatening,

(3) results in disability (i.e., dysfunction that interferes with daily living activities),

(4) results in potential disability,

(5) requires hospitalization or prolongation of existing hospitalization for treatment,

(6) is serious on the lines of (1) to (5) above, or

(7) is a congenital disease or anomaly in the offspring.

Of note, “hospitalization” in (5) above does not include hospitalization or prolonged hospitalization only for repeat testing, follow-up, or implementation of therapy or testing planned before the study to be performed during the study (e.g., pre-planned surgery or testing). However, any new event occurring during such hospitalization will be handled as an adverse event.

1) Reportable serious adverse events：Reporting is required for all serious adverse events occurring during the study period, as well as serious adverse events occurring after the end (or discontinuation) of the study and suspected to be related to the UV irradiation.

2) When an unexpected serious adverse event or malfunction related to the study occurs and a direct causal relationship with the study cannot be denied, the principal investigator will report to the head of the research institution and cooperate with submission of the “Unexpected Serious Adverse Event Report Form” to the Minister of Health, Labour and Welfare by the head of the research institution.

# **11．Completion, termination, and suspension of the trial**

・Criteria for discontinuation or suspension of the study

1. Case registration will be suspended if any serious adverse event that could lead to death or mortality and that a relationship with UV irradiation cannot be denied is observed.
2. If any erythema is observed after 50 mJ/cm^2^ UV irradiation in the first step of this study (8. (1)), the study will be discontinued.
3. When any of the following information is obtained, the principal investigator will decide discontinuation or suspension of the entire study based on the judgement by the principal investigator or discussion with the head of the research institution. If the ethical committee instructs to cancel or suspend the study, investigators will follow the instructions promptly.

1) When there is a problem with the safety and effectiveness of UV irradiation.

2) When there is a problem with the safety or effectiveness of the protocol treatment.

3) When there is a cancellation recommendation from the regulatory authority.

4) When there is a change in the development policy of the principal investigator.

5) When other situations arise that require some or all of the study to be discontinued or suspended.

・Procedures for discontinuing or suspending the entire study

1. When the study at a research institution is discontinued or suspended, the principal investigator will promptly notify the fact of discontinuation/suspension to the study subjects and guarantee appropriate procedures such as switching to an appropriate treatment.
2. When the study at a research institution is discontinued or suspended, the principal investigator will promptly notify this to the head of the research institution in writing.
3. When the principal investigator has decided discontinuation or suspension of the study, the principal investigator will notify the details of that and the reason in writing to the head of the research institution.

# **12．Study implementation period**

June 15, 2017 to May 15, 2018 (Final enrollment date: May 15, 2018)

Enrollment period: 11 months. Study period: 12 months.

# **13. Subject analysis (sub-group for effectiveness evaluation) and method of statistical analysis (primary/secondary analysis)**

A summary of the planned statistical analysis of this study is provided below. Details of the planned statistical analysis will be described in the statistical analysis plan. The summary of the planned statistical analysis described in this protocol may be changed in the statistical analysis plan. If the definition or analysis method for the primary endpoint is changed, the protocol will be amended.

1. Analysis populations

（1） Full analysis set (FAS)

The full analysis set (FAS) will consist of all subjects enrolled in the study, who have efficacy data available, excluding those without baseline data or significant protocol violations (e.g., absence of informed consent, enrollment outside the contract period).

（2）Per protocol set (PPS)

The per protocol set (PPS) will consist of the subjects in the FAS excluding those with any of the following significant protocol violations involving the study method, concomitant therapy, etc.:

- violation of the inclusion criteria
- violation of the exclusion criteria
- violation of prohibited concomitant medications
- violation of prohibited concomitant therapies

（3）Safety analysis set

The safety analysis set will consist of the subjects enrolled in this study who received any UV irradiation.

2．Target sample size and rationale

Target sample size: 20

This exploratory study will be conducted to gather information to properly design new clinical trials with patients. For this reason, the target number of cases was set in order to collect a wide range of efficacy and safety indicators. Theoretically, it is assumed that the possibility that 222 nm UVC will not cause erythema in the irradiated area is almost 100%. It is thought that this irradiation method will be judged to be appropriately safe when the one-sided 95% confidence interval lower limit is higher than 75%. In this study, the number of enrollment subjects will be gradually increased from 2 subjects to 20 subjects in total. If erythema does not appear in all 20 participants, the one-sided 95% confidence interval lower limit will be 83.9%. Since this test will be conducted as an exploratory one, when considering the implementation of the next phase of the test, the primary and secondary evaluation items will be comprehensively judged.

3．Items and plan of the statistical analysis

The analysis will be performed after the completion of UV irradiation and sampling in all subjects. For all efficacy endpoints, the FAS will be used in the primary analysis, while the PPS will be used in a reference analysis. Safety will be analyzed using the safety analysis set.

(1) Safety analysis

1) Presence or absence of erythema

The erythema non-occurrence rate and its 95% confidence interval for each dose will be calculated.

2) Examination of DNA damage

The amount of CPD produced in the UV irradiation group and non-irradiation group will be compared using a paired *t*-test. If the amount of CPD produced is significantly higher in the irradiated group than in the non-irradiated group, it will be determined that there is DNA damage due to UV irradiation. The hypothesis testing will use a significance level of two-sided 5%, with the calculation of the two-sided 95% confidence interval.

3) Frequency of adverse events

A summary table will be prepared for the endpoint. For estimation of the proportion, the exact two-sided 95% confidence interval for a binomial distribution will be calculated per group. As necessary, Fisher’s exact test will be used for inter-group comparison.

(2) Efficacy analysis

1) Primary analysis

Bacterial detection frequency from skin swabs will be compared using the Χ^2^ test. The hypothesis testing will use a significance level of two-sided 5%, with the calculation of the two-sided 95% confidence interval.

2) Secondary analysis

The secondary efficacy endpoints will be analyzed for discussion to supplement the primary analysis results. No multiplicity adjustment will be performed in the analysis of secondary efficacy endpoints. The hypothesis testing will use a significance level of two-sided 5%, with the calculation of the two-sided 95% confidence interval.

(3) Interim analysis

No interim analysis is planned in this study.

(4) Independent data monitoring committee (IDMC)

An IDMC will not be established in this study.

(5) Final analysis

The final analysis will be performed after data from the subjects have been obtained and locked after the end of the follow-up period. The responsible biostatistician will prepare the “statistical analysis report” and submit it to the principal investigator.

# **14．Quality assurance of the trial**

1. Quality control

Monitoring of the study will be performed to periodically check whether this study is conducted safely in accordance with the protocol and whether the data are properly collected.

(1) Appointment of the responsible monitor and other monitors

The principal investigator will appoint the responsible monitor and other monitors for this study.

(2) Implementation of study monitoring

1) Monitoring on subject data

The monitor will check the CRFs (accumulated database) submitted to the data center throughout the study period. As necessary, direct reviews of source documents (e.g., informed consent forms, medical charts, CRFs) and off-site monitoring will be performed at Kobe University Hospital. The items to be checked at monitoring are specified in the “Written procedure for implementation of study monitoring”.

2) Monitoring on non-subject data

The monitor will perform study monitoring before, during, and after the end (discontinuation or suspension) of the study. The items to be checked at monitoring are specified in the “Written procedure for implementation of study monitoring”.

2．Quality assurance

In this exploratory study, performed in a small number of patients, no auditing is planned.

# **15．Consideration of human rights and safety/disadvantages to the subjects**

1) Consideration of human rights (privacy protection)

The protection of the subject's privacy should be considered when preparing the CRF and handling the subject's data. That is, the name and initials of the subject are not to be used but are specified by the subject identification code. The principal investigator creates a correspondence table (subject identification code table) of the medical record number, patient name, date of birth, and patient identification code set in the facility. The subject identification code table is kept by the principal investigator.

2) Consideration of safety and disadvantages

If a subject suffers health injury related to the study, the sub-investigator will perform appropriate action and treatment. It should be described that information necessary for conducting clinical research safely is collected and reviewed, and the research protocol could be changed as necessary.

# **16．Expenses for the subjects**

1) Compensation for health injury

If a subject suffers health injury related to this study, the sub-investigator will perform appropriate action and treatment. For compensation of health injury, the “Ethical Guidelines for Medical and Health Research involving Human Subjects” will be followed. Thus, a clinical study liability insurance policy will be taken out to be able to compensate for death or permanent disability (grade 1 to 14 permanent disability) resulting from this study. For other health injury, the tests, treatments, and other necessary procedures will be performed within the range of medical services covered by the subject’s health insurance.

2) Participation in liability insurance

In preparation for liability, the principal investigator or sub-investigators will participate in liability insurance.

# **17．Compliance with ethical guidelines including the Declaration of Helsinki**

This study will be conducted in compliance with the Ethical Guidelines for Medical and Health Research involving Human Subjects (Notice of Ministry of Health, Labour and Welfare. December 22, 2014), the Declaration of Helsinki, and Kobe University Conflicts of Interest Management Guideline.

# **18．Retention of recorded documents**

In accordance with the “Guideline on the study data storage period at Kobe University Graduate School of Medicine etc.”, the study representative will retain the study-related important documents (e.g., copies of the application forms submitted to the ethics review committee, notification documents from the head of the research institution, copies of various application forms and reports, documents for data disclosure, and other documents or records supporting the information used in the study including the data correction log and description in notebooks) in a lockable place, either until 10 years after study discontinuation/completion or 10 years after the date of publication of the study results in the literature etc., whichever is later. Thereafter, the documents will be destroyed after subject individuals are made non-identifiable.

At each collaborative research implementing entity, the study data etc. will be appropriately retained for the duration required by the rules of the collaborative research implementing entity.

# **19．Registration of research program**

Based on counsel by the International Committee of Medical Journal Editors (ICMJE), this research program will be registered to University Hospital Medical Information Network-Clinical Trial Registry (UMIN-CTR) or US national library of medicine clinical trial registration open website (ClinicalTrials.gov) by the start of this research.

# **20．Research organization**

1．Principal investigator and sub-investigators

（Name） （Department） （Position） （TEL）

・Kobe University Hospital

○Takahiro Niikura Orthopaedic Surgery Lecturer +81-78-382-5985

Ryosuke Kuroda Orthopaedic Surgery Professor +81-78-382-5985

Keisuke Oe Orthopaedic Surgery Assistant Professor +81-78-382-5985

Tomoaki Fukui Orthopaedic Surgery Medical staff +81-78-382-5985

(○：Principal investigator)

・Collaborative research institute：Ushio Inc.

Principal investigator：Tatsushi Igarashi, Business promotion department、Project leader

Address：2-6-1, Ohtemachi, Chiyoda-ku, Tokyo 100-8150 JAPAN

TEL：+81-3-6328-3447

・Outsourcing organization：Cosmo Bio Co., Ltd.

Address：2-2-20, Toyo, Koto-ku, Tokyo 135-0016, Japan

TEL：＋81-3-5632-9600

FAX：＋81-3-5632-9613

＜Study secretariat＞

Department of Orthopaedic Surgery, Kobe University

Dr. Takahiro Niikura (Representative), Dr. Keisuke Oe, Dr. Tomoaki Fukui

Address：7-5-2, Kusunoki-cho, Chuo-ku, Kobe, Hyougo 650-0017 JAPAN

TEL：+81-78-382-5985、FAX：+81-78-351-6944

E-mail：[tniikura@med.kobe-u.ac.jp](mailto:tniikura@med.kobe-u.ac.jp) (Dr. Takahiro Niikura)

2．Protocol creation

Responsible：Dr. Takahiro Niikura, Department of Orthopaedic Surgery, Lecturer

Staff：Dr. Tomoaki Fukui, Department of Orthopaedic Surgery, Medical Staff

3．Data management

Responsible：Dr. Takahiro Niikura, Department of Orthopaedic Surgery, Lecturer

Staff：Dr. Tomoaki Fukui, Department of Orthopaedic Surgery, Medical Staff

4．Responsible monitor

Dr. Hanako Nishimoto, Division of Rehabilitation, Medical staff

# **21．Disclosure of research funding sources and COI status of researchers**

There are no interests, monetary and individual relationships that affect the outcome of the research in the planning, implementation, and publication of this study. This study is an independent clinical study and is funded by the research expenses of the department to which the principal investigator belongs. This study is not based on financial support from Ushio inc. which produces 222nm UVC irradiator used in this study and other organizations. However, 222nm UVC irradiator, medical equipment used in this study, will be provided free by Ushio Inc.

# **22．Change of study protocol**

Changes (revisions) of the study protocol, informed consent form will be made after obtaining approval from the ethics committee of the medical institution in advance.

# **23．Publication of research results**

The contents of this research will be presented at the appropriate international and domestic conference and be published in a peer reviewed journal after the study completion.

# **24．References**

1. Sumiyama Y, et al.: Current status and problems of surgical infection control. Geka. 67:125-131, 2005. (Japanese)

2．Skaramm I, et al.: Surgical site infections in orthopaedic surgery demonstrate clones similar to those in orthopaedic *Staphylococcus aureus* nasal carriers. J Bone Joint Surg Am. 96(11):882-888, 2014.

3. Nishikawa K, et al.: Where does the surgical site infection (SSI) originate from? Influence of surgical field contamination to the SSI (wound). Jpn J Gastroenterol Surg. 41(1):12-21, 2008. (Japanese)

4. Wenzel RP, et al.: The significance of nasal carriage of and the incidence of postoperative wound infection. J Hosp Infect. 31(1):13-24, 1995.

5. Kreusch S, et al.: UV measurements in microplates suitable for high-throughput protein determination. Anal Biochem. 313:208-15, 2003.

6. Buonanno M et al.: Germicidal efficacy and mammalian skin safety of 222 nm UV light. Radiat Res. 187(4):483-491, 2017.

7. K. Narita, et al.: Insight into disinfection effect and healing process by irradiating 222 nm UVC light on drug resistant bacteria MRSA infected with mouse wounds. 178:10-18, 2018.

8. Igarashi T. Ushio Inc. Private letter. Mar. 1. 2017.

9. Woods JA, et al. The effect of 222 nm UVC phototesting on healthy volunteer skin: a pilot study. Photodermatol Photoimmunol Photomed. 31(3):159-66, 2015.
