# Supplementary material for: Exploratory clinical trial on the safety and bactericidal effect of 222-nm ultraviolet C irradiation in healthy humans
Source: PLoS One. 2020 Aug 12;15(8):e0235948. doi: 10.1371/journal.pone.0235948 (PMC7423062; doi:10.1371/journal.pone.0235948)
Supplement: S3 File — (PDF) [file pone.0235948.s004.pdf]

健常者を対象とした 222nm UVC 照射の

安全性および殺菌作用に関する探索的臨床試験

試験実施計画書

第 2.1 版　　2017 年 10 月 11 日

試験代表者

氏名：新倉　隆宏

所属：神戸大学医学部附属病院　整形外科

TEL：078-382-5985

FAX：078-351-6944

E-mail：tniikura@med.kobe-u.ac.jp

試験事務局

事務局名:神戸大学医学部附属病院　整形外科

事務局住所：神戸市中央区楠町 7-5-2

TEL：078-382-5985

FAX：078-351-6944

E-mail：tniikura@med.kobe-u.ac.jp

目次

1. 背景 ... 3
2. 試験の目的と必要性 ... 5
3. 試験機器の概要 ... 5
4. 研究対象者 ... 6
5. 研究対象者に説明して同意（インフォームドコンセント）を得る方法 ... 7
6. 試験の方法 ... 8
7. 評価項目 ... 9
8. 観察および検査項目 ... 10
9. 中止基準 ... 12
10. 有害事象発生時の取扱い ... 13
11. 試験の終了・中止・中断 ... 14
12. 試験実施期間 ... 15
13. 解析対象（有効性評価の部分集団）、および統計解析方法（主要解析・副次的解析） ... 15
14. 臨床試験実施のための品質保証 ... 17
15. 研究対象者の人権および安全性・不利益に対する配慮 ... 17
16. 研究対象者の費用負担 ... 18
17. 倫理指針およびヘルシンキ宣言等への対応 ... 18
18. 記録文書等の保存 ... 18
19. 研究計画の登録 ... 18
20. 研究組織 ... 18
21. 研究資金源および各研究者の COI 状態の開示 ... 19
22. 実施計画書等の変更 ... 20
23. 研究結果の公表 ... 20
24. 文献リスト・参考資料 ... 20

1．背景

現代の医療において外科的治療が占める割合は大きく、それを必要とする患者は数多い。複数の診療科で国内外を問わず多様な手術治療が行われているが、様々な合併症が発生する危険性があり、その中で最も重大なものの一つが周術期感染である。ひとたび感染が成立すれば治療に難渋することとなり、患者・医療者ともに多大な負担を強いられることとなる。近年、ガイドラインに基づいた抗生剤の投与などで周術期感染を予防する対策がとられるようになったにも拘らず日常診療において周術期感染はしばしば発生しており、依然として更に有効な予防策の確立が必要である。

周術期感染の発生原因として、環境因子、患者因子、細菌因子などが考えられるが[1]、細菌因子すなわち周術期感染の起因菌は、患者の皮膚や鼻腔粘膜の常在菌のような内因性のものと落下細菌のような外因性のものに分けられる。両者のどちらがより重要であるかのコンセンサスは得られていないが、内因性の細菌感染の関与を強調する多くの報告がある[2,3,4]。手術前には十分に消毒された術野であっても、手術中に皮膚常在菌が繁殖することによる手術創の汚染を持続的に制御する必要がある。

一方、古くから紫外線照射には殺菌効果があることが知られている。紫外線照射により細菌の DNA、RNA の隣接した塩基が二量体化されることで、細菌の増殖は停止し、死滅する。特に 200～280nm と波長の短い紫外線 C 型（UVC）は、DNA の吸収係数が高いため殺菌効果が高く、殺菌灯として使用されている。また、その殺菌効果を応用し、米国、カナダでは 254nm の UVC を照射する機器が感染を伴った創部の治療に対する治療器として認可を受け臨床で使用されている。しかし、UVC の照射には細胞毒性があり悪性腫瘍の発生などの危険性が懸念され、より安全な方法が望まれる。

254nm UVC は皮膚に照射すると、角質を通過して表皮まで到達するため、表皮細胞に影響を及ぼす。一方 222nm UVC は、タンパク質の吸収係数が高く、表皮の最表層にある角質層までしか到達しないため皮膚の細胞群へは影響を及ぼさず非常に安全性の高い UVC と考えられる。また角質がない創傷へ照射しても、ヒトの細胞が細菌の 1μm 以下に対し、5-25μm と大きく、222nm UVC はヒトの細胞核まで到達しないため、DNA へのダメージがなく、安全と考えられる。このことから、より多量の紫外線照射が可能である。

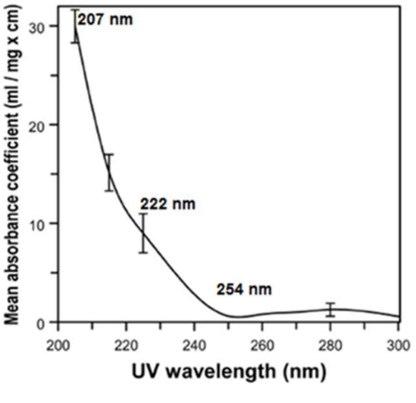

図 1: 8 種類タンパク質の UV 吸収係数。200nm 付近 UV は生体内 0.3μm で半減する。250nm 付近 UV は 3μm で半減 する[5]。

一方　同一照射量での 222nmUVC と 254nmUVC 殺菌能力は図 2 に示すように同等である。

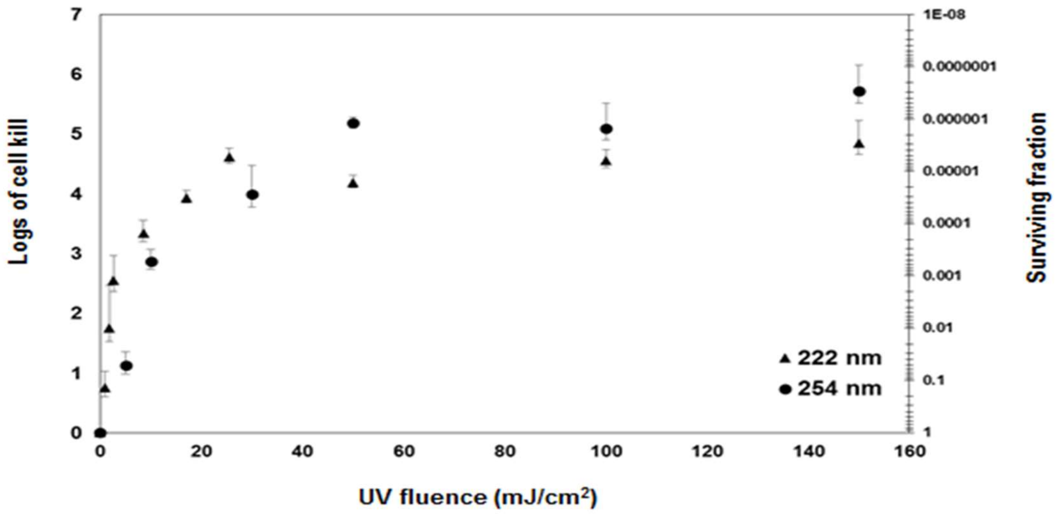

図 2: 222nmUVC および 254nmUVC のフルーエンスと MRSA 殺菌率の関係 [6]

これらのことから 222nmUVC は 254nmUVC に対し、殺菌性の向上が期待できる。

安全性動物実験はコロンビア大 Buonanno M ら、弘前大成田らが行っている。Buonanno M らは、ヘアレスマウスに 254nm、222nm を 164mJ/cm^2 照射した。細胞核(DNA)の UV ダメージマーカーとしてシクロブタン型ピリミジンダイマー(CPD)、6-4photoproducts（6-4PP）に着目して測定したが、254nm の照射後のみ、CPD,6-4PP の生成を認め、DNA のダメージが確認された [6]。

成田らはマウスの正常皮膚上と表皮がない創傷に 222nmUVC を 500 mJ/cm^2 照射し、細胞核の変幻性を CPD をマーカーとして調べ、254nmUVC 照射の場合と比較した [7]。
実験結果は以下のとおりであった。

1) 正常皮膚に 500mJ/cm^2、222nmUVC を照射後、CPD は検出されなかった。254nmUVC 照射の場合は、角化細胞の 60%で CPD が検出された。（図 3）
2) 創傷に 500mJ/cm^2、222nmUVC を照射後、CPD は検出されなかった。254nmUVC 照射の場合は、1 時間後では線維芽細胞の 80%から CPD が検出された。（図 4）

人への照射について、現在、シンガポール国立大学において、褥瘡のバクテリアマネージメントに関しての Human　POC がスタートしている [8]。使用されている装置は、今回用いるウシオ電機により開発されたものと同装置である。1 回の照射量は 500mJ/cm^2、2 日に 1 回 2 週間の照射が行われている。現在、2 名の患者の照射が終了しており、照射後、バクテリア量の減少が認められた。創傷周辺の平常皮膚は照射後、紅斑は認められていない。

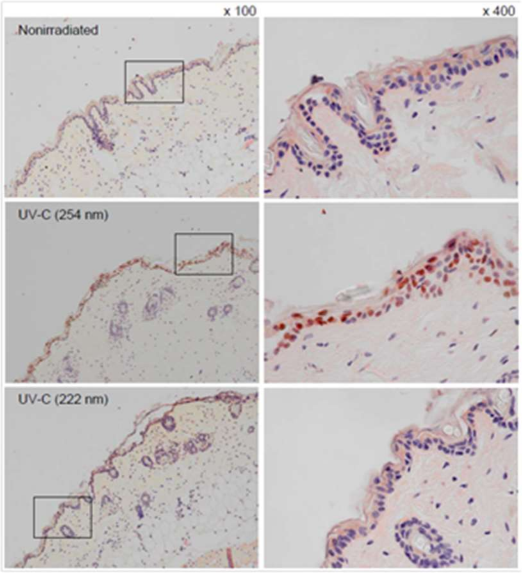

図 3: 222nmUVC および 254nmUVC を正常皮膚に 500 mJ/cm^2 照射後、Anti - CPD Ab 染色した組織切片の顕微鏡写真。赤茶色の細胞は抗 CPD 抗体との反応による結果である。

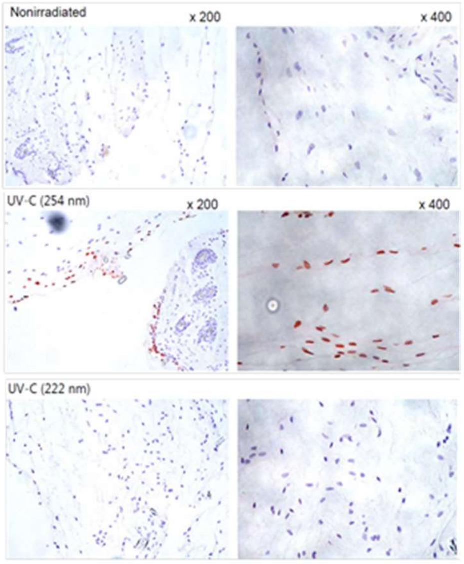

図 4：222nm 光および 254nm 光を創傷に 500 mJ/cm^2 照射後 Anti - CPD Ab 染色した組織切片の顕微鏡写真。赤茶色の細胞は抗 CPD 抗体との反応による結果である。

Woods JA らによって、222nm　KrCl エキシマランプで、ヒトの正常皮膚に対する照射実験も報告されている[9]。この報告では、一部の対象者において照射された範囲の皮膚組織に CPD の生成が認められたが、222nm よりも波長の長い UVC も混在していたために起こったと結論付けられている。

我々は、222nm の UVC 照射により周術期の感染率を劇的に改善できる可能性があると考え、ウシオ電機により開発された 222nm UVC 照射器を手術野に照射する機器を臨床に応用することを考えた。本機器は先に挙げた Woods らで報告された照射器と違い、シンガポール国立大学での使用装置と同様、フィルターを使用することで 222nm 以外の周波数の UVC を除去することでより特異的に効果を評価することのできる機器となっている。

2. 試験の目的と必要性

健常者ボランティアを対象として、222nm UVC照射器による紫外線照射の安全性を確認し、またその皮膚殺菌作用を検討すること。

3 試験機器の概要

222nm UVC 照射器。詳細は添付文書を参照のこと。

1）試験機器情報

222nm UVC 照射器：株式会社ウシオ電機

2）予期される有害反応および医療機器の場合は予期される不具合

理論上､有害反応は発生する可能性は極めて低いと考えられるが､222nm UVC照射器での紫外線照射によるヒトの皮膚障害の出現及び皮膚細胞内の遺伝子異常が発生することを否定する evidence が存在しない。

4. 研究対象者

以下の適格基準を全て満たし、かつ以下の除外基準のいずれにも該当しない方を、本研究の対象とする。

1） 選択基準：

1）同意取得時に、年齢が 20 歳以上 80 歳未満の方
2）性別：不問
3）本試験への参加について、本人の自由意思による文書同意が得られた方

2）除外基準：

1）アレルギー等薬剤過敏体質の方
2）背部の紫外線照射予定部位に皮膚炎や外傷などの異常がある方
3）背部の紫外線照射予定部位に外用薬や貼付剤を常用している方
4）背部の紫外線照射予定部位に入れ墨のある方
5）妊婦および妊娠している可能性のある患者、または授乳中の方
6）４ヶ月以内に他の臨床試験に参加した方
7）その他、本試験の担当医師が不適当と判断した方

設定理由
1）、2）被験者の安全性を確保するために設定した。
2）-4）本試験機器での紫外線照射による変化を評価するのに支障をきたす可能性が考えられるために設定した。
5）本試験機器使用の妊婦および胎児への安全性が確認されていないために設定した。
6）臨床研究へ参加する被験者への倫理的配慮を考慮し設定した。
7）研究責任医師が前述以外の全般的要因を勘案し、被験者の安全性確保を配慮して本研究の参加の可否を判断できる余地を残すため設定した。

3）目標対象者数
20 名

4）対象者組み入れの手順

安全性を担保するため、まず 2 例で紫外線照射と照射後 24 時間での紅斑有無の判定を行い、最低照射量である 50mJ/cm^2 の照射で紅斑が出現するという状況

でなければ、次は 4 例で行い、その後 6 例、8 例と段階的に組み入れ症例数を増加させていく予定である。（下記 8. （ 1 ）の＜第 1 段階＞）1 例でも、50mJ/cm^2 の照射で紅斑が出現する被検者が出た場合、試験を中止する。

20 例の対象者が組み入れされた後、下記 8. （1）の＜第 2 段階＞を行う。

5. 研究対象者に説明して同意（インフォームドコンセント）を得る方法

試験開始前に、試験担当医師は被験者候補に対し下記の内容について文書および口頭で十分説明する。説明にあたっては、当院介入研究倫理審査委員会で承認された同意説明文書を用いる。被験者候補には質問する機会、および同意するかどうかを判断するための十分な時間を与え、本試験の内容を良く理解したことを確認した上で、自由意思による同意を得る。被験者候補から同意が得られる場合は、被験者候補からの同意文書等への署名または記名捺印、および同意年月日の記入を得る。説明者（試験責任医師または試験分担医師）も同意文書等に署名または記名捺印し、説明年月日を記入する。被験者および説明者の署名または記名捺印と年月日の記入された同意文書等の原本は試験事務局に保管し、その写しを被験者および代諾者に渡す。

① はじめに（この試験が研究を伴うこと）
② この臨床試験の目的
③ この臨床試験の方法について
④ この試験の参加予定者数
⑤ 期待される効果について
⑥ 予想される副作用と危険性について
⑦ この試験中に、あなたの健康に被害が生じた場合について
⑧ 遺伝子解析などの情報の開示と期待される利益および予想される不利益について
⑨ この試験に関わる費用について
⑩ 同意しない場合でも不利益は受けないこと
⑪ 同意後の撤回について
⑫ 試料等の保存及び使用方法並びに保存期間
⑬ 治療計画書等の開示について
⑭ プライバシーの保護について
⑮ 個人情報等の閲覧について
⑯ 知的財産権の帰属について
⑰ 当該研究に係る資金源、利益相反について
⑱ あなたに守っていただきたいこと
⑲ 問い合わせ窓口
⑳ 研究機関、研究責任者について

6. 試験の方法

以下の項目について記載する。

1）試験の種類・デザイン

探索的臨床試験

2）試験のアウトライン

本試験は、健常者を対象に、222nm UVC 照射器の安全性を確認し有効性を検証するための探索的臨床試験である。

試験スケジュールの概略を図 5 に示す。

<u>同意取得後試験開始</u>

＜第 1 段階＞

UVC 照射

	照射直前	24 時間
紅斑<br>チェック	○	○

→ <u>第 2 段階での照射量決定</u>

＜第 2 段階＞

UVC 照射

	照射直前	5 分	30 分	1 時間
スワブ	○	○	○	
生検		○		

→ <u>7 日以内にゲノム DNA 採取し CPD 濃度測定</u>

図 5. 試験のアウトライン

3）研究対象者の試験参加予定期間

予定登録患者数：20名。

登録期間：11 か月。追跡期間：試験開始後約 1 か月間（CPD テスト結果判明まで）。総研究期間：12 か月。

4）紫外線照射の方法

健常者の背部、第 3 胸椎から第 12 胸椎の範囲で肉眼的に皮膚の異常が無い部分に紫外線を照射する。本研究は 222nm UVC 照射器の安全性を確認する目的があり、理論上紅斑や皮膚細胞の DNA 障害が発生しないことを想定していることから、最大量の照射が必要と考えた。254nm UVC の最小紅斑量が 10 mJ/cm^2 であることを考慮し、十分な照射量として 500mJ/cm^2 を最大照射量と設定した。

もし 500mJ/cm2 以下で紅斑の出現が認められた場合には、紅斑が出現しない最大照射量が、今後の試験での推奨照射量となると考えられる。

5）紅斑の判定方法

非照射部位と比較し、かろうじて識別できる変化が照射部位に認められれば紅斑ありと判定する。2 名以上で判定し、1 名でも紅斑ありと認めた場合に紅斑ありと判定する。照射部位と非照射部位を同一写真内に撮影し、データ保存する。

6）併用薬（療法）に関する規定

① 併用薬（療法）: 特に併用薬（療法）に関する規定は設けない。

② 併用禁止薬（療法）: 本研究に使用するもの以外の紫外線照射装置の使用は禁止する。

③ 併用制限薬（療法）: 背部への塗布剤や貼付剤の使用は照射 2 日前より使用しないように制限する。

④ 併用注意薬（療法）: 特に併用薬（療法）に関する規定は設けない。

7）試料等の保存および他機関等の試料等の利用

試験責任（分担）医師は、試験等の実施に関する試料（生体試料、標本）や装置等は、本研究の結果の最終の公表について報告された日から 5 年間、試験などの実施に関するデータは 10 年間原則として保存し、その後は個人を特定できない状態にして廃棄する。

本研究で得られた研究対象者の試料・データは、同意を受ける時点では特定されない将来の研究のために用いる可能性がある。その場合には、別途研究対象者に説明した上で実施する。

・試料・データの提供元：神戸大学医学部附属病院整形外科

・試料・データの提供先：ウシオ電機株式会社、コスモ・バイオ株式会社

7. 評価項目

1）主要評価項目（Primary endpoint）:

・500mJ/cm^2 以下の紫外線照射終了後 24 時間での皮膚紅斑の有無。

2）副次的評価項目（Secondary endpoint）

1. 紫外線照射による皮膚常在菌殺菌効果の有無。
2. 紫外線照射後の皮膚細胞内での DNA 損傷の有無。
3. 有害事象発生の有無。

8. 観察および検査項目

（1）試験実施中に収集予定の観察および検査項目

1　同意取得時

・ 研究対象者識別コードを確認し、性別・生年月日・身長・体重・合併症・既往歴の測定・聴取を行い、適格性を評価する。

＜第 1 段階＞

2　照射前

・ 被検者背部に 6 か所（E1、E2、E3、E4、E5、E6。1 か所 10×10mm。）の照射範囲を指定する。照射量は照射範囲に左右されないので厳密な面積の設定は不要である。被験者背部に紫外線照射を行わない範囲（N、10×10mm）も指定する。（図 6）

3　紫外線照射

・ E1、E2、E3 にそれぞれ 50、100、200mJ/cm^2 の UVC を照射する。照射量は照射機器自体で測定することが可能である。照射に必要な時間は 50mJ/cm^2 で約 10 秒、100mJ/cm^2 で約 20 秒、200mJ/cm^2 で約 40 秒、の予定である。（図 6）

	UVC (+) 50mJ/cm^2	UVC (+) 100mJ/cm^2	UVC (+) 200mJ/cm^2	UVC (-)
紅斑	**E1**	**E2**	**E3**	**N**

図 6　照射部位と照射量①

4　照射後 24 時間

・ N と比較し E1、E2、E3 の紅斑の有無を評価する。非照射部位および照射部位の写真撮影し保存する。24 時間後で紅斑が認められなければ 5 に進む。紅斑が認められた場合、紅斑を生じる最小の照射量を最小紅斑量（MED:Minimum Etythema Dose）とし、第 2 段階の UVC 照射量を下記 7 の通り決定する。

5　紫外線照射

・ E4、E5、E6 にそれぞれ 300、400、500mJ/cm^2 の UVC を照射する。照射に必要な時間は 300mJ/cm^2 で約 60 秒、400mJ/cm^2 で約 80 秒、500mJ/cm^2 で約 100 秒、の予定である。（図 7）

	UVC (+) 300mJ/cm^2	UVC (+) 400mJ/cm^2	UVC (+) 500mJ/cm^2	UVC (-)
紅斑	**E4**	**E5**	**E6**	**N**

図 7　照射部位と照射量②

6　照射後 24 時間

・ N と比較し E4、E5、E6 の紅斑の有無を評価する。非照射部位および照射部位の写真撮影し保存する。紅斑が認められた場合 MED を決定し、第 2 段階の UVC 照射量を下記 7 の通り決定する。全 20 症例の MED 決定が終了した後に第 2 段階へ移る。

7　第 2 段階の試験における照射量の決定

・ 全 20 症例の中で最も低い MED を基準に第 2 段階における照射量を図 8 のように決定する。

全症例のうち最も低い MED	第 2 段階での照射量
MED なし（500 mJ/cm^2 で紅斑なし）	500 mJ/cm^2
500 mJ/cm^2	400 mJ/cm^2
400 mJ/cm^2	300 mJ/cm^2
300 mJ/cm^2	200 mJ/cm^2
200 mJ/cm^2	100 mJ/cm^2
100 mJ/cm^2	50 mJ/cm^2
50 mJ/cm^2	第 2 段階を行わず中止

図 8　第 2 段階での照射量の決定

＜第 2 段階＞

8　紫外線照射

・ 被検者背部に 3 か所（S5、S30、B）の照射範囲（40×40mm）を指定し、7

で決定した照射量の UVC を照射する。（図 9）

- N0 の表面をスワブ（エルメックス社、Pro-media st-25）で擦過し、培養検査へ提出する。

	UVC (+)	UVC (-)
スワブ：照射前		**N0**
スワブ：照射後 5 分	**S5**	**N5**
スワブ：照射後 30 分	**S30**	**N30**
生検：照射後 1 時間以内	**B**	**NB**

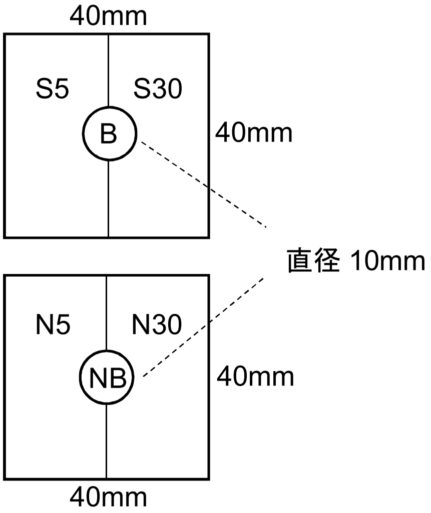

図 9. 照射部位と照射量③

9　照射後 5 分

- S5、N5 の表面をスワブで擦過し、培養検査へ提出する。

10　照射後 30 分

- S30、N30 の表面をスワブで擦過し、培養検査へ提出する。

11　照射後 1 時間以内

- B、NB に局所麻酔を行い直径 3mm の生検用トレパン（KAI メディカル株式会社、BP-30F）を用いて皮膚組織を採取し速やかに冷凍保存する。→12 へ。

12　生検組織採取後 7 日以内

- 生検組織からゲノム DNA を採取する。QIAamp Blood Kit （QIAGEN, Cat. No. 51104 or 51106）を用いて行う。ゲノム DNA の濃度測定を行う。
- ゲノム DNA サンプルを株式会社コスモ・バイオに送付し、紫外線照射による DNA 損傷の指標となる CPD（cyclobutane pyrimidine dimer；シクロブタン型ピリミジン二量体）を測定する。照射群と非照射群で CPD 生成量を群間比較し、統計学的有意差をもって CPD 生成量が増加していれば DNA 損傷ありと判定する。

9. 中止基準

研究責任者または研究分担者は、以下に示す理由で試験継続が不可能と判断した場合には、試験を中止し、中止・脱落の日付・時期、中止・脱落の理由、経過をカルテなら

びに症例報告書に明記するとともに、中止・脱落時点で必要な検査を行い有効性・安全性の評価を行う。

① 研究対象者から試験参加の辞退の申し出や同意の撤回があった場合
② 登録後に適格性を満足しないことが判明した場合
③ 合併症の増悪により試験の継続が困難な場合
④ 有害事象により試験の継続が困難な場合
⑤ 妊娠が判明した場合
⑥ 試験全体が中止された場合
⑦ その他の理由により、医師が試験を中止することが適当と判断した場合

10. 有害事象発生時の取扱い

試験責任医師および分担医師は、有害事象等を研究対象者から聴取し、有害事象を認めた時は、直ちに適切な処置を行うとともに、症例報告書に齟齬なく記載する。試験責任医師および分担医師は、有害事象について、紫外線照射との関連性に係わらず、症状が消失するまで、そうでない場合（永続的・不可逆的な有害事象等）には、観察された変化について十分な説明がつくまで、すべての研究対象者を追跡調査する。すべての有害事象を症例報告書に記録する。有害事象の名称、重症度、重篤性、紫外線照射との因果関係（関連なし、関連あり）について記録する。また最終診察（紫外線照射および観察終了後に発現した有害事象含む）では、紫外線照射に関する処置、転帰についても記録する。DNA 損傷が認められた場合には再度同じ照射量での照射を行い、照射後 48 時間で生検を行い、CPD を測定する。CPD 生成量に照射群、非照射群で有意差がある場合は、照射後 3 か月間、照射部位の異常が無いか観察する。

有害事象等の追跡期間は、有害事象が回復するまで、又は試験責任医師および分担医師がこれ以上の追跡は不要と判断するまでの期間とする。

・重篤な有害事象の報告

重篤な有害事象とは、次のいずれかに該当するものとする。

（1） 死亡
（2） 死亡につながるおそれのあるもの
（3） 障害（日常生活に支障をきたす程度の機能不全の発現）
（4） 障害につながるおそれのあるもの
（5） 治療のために病院又は診療所への入院又は入院期間の延長が必要とされるもの
（6） （1）～（5）までに掲げる症例に準じて重篤であるもの
（7） 後世代における先天性の疾病又は異常

なお、（5）の「入院」には、再検査、追跡調査のための入院又は入院期間の延長、及び試験前より予定していた療法又は検査を試験中に実施することのみを目的とし

た入院（予定手術や検査等）は含まれない。（ただし、その入院中に新たに発生したものは有害事象として取扱う。）

1）報告の対象となる有害事象：試験期間中の全ての重篤な有害事象および試験終了（中止）後にとの関連性が疑われる重篤な有害事象について報告する。

2）研究責任者は、重篤な有害事象の発生を認めた時は、速やかに研究機関の長に報告するとともに、多施設共同試験において紫外線照射との因果関係を否定できない場合、所属研究機関の長から他の医療機関の研究責任者に研究代表者を通じて報告する。報告は第一報（緊急報告）および第二報（詳細報告）とする。

3）研究責任者は、臨床研究に関連する予期しない重篤な有害事象および不具合等が発生した場合であって当該研究との直接の因果関係が否定できないときには、速やかに研究機関の長に報告するとともに、研究機関の長による厚生労働大臣へ「予期しない重篤な有害事象報告」の報告ならびに公表について協力する。

11. 試験の終了・中止・中断

・試験の中止・中断基準

(1) 紫外線照射との関連性が否定できない死亡または死亡につながるおそれのある重篤な有害事象が 1 例でも認められた場合、症例登録を一時中断する。

(2) 試験の第 1 段階（上記 8.（1））において、1 例でも 50mJ/cm^2 の照射で紅斑が出現した場合、試験を中止する。

(3) 試験責任医師は、下記のいずれかに該当する場合、試験責任医師の判断もしくは実施医療機関の長等と協議の上、試験全体の中止または中断について決定する。介入研究倫理審査委員会から中止または中断を指示された場合には、速やかに指示に従う。

1） 紫外線照射の安全性・有効性に問題が生じた場合
2） プロトコル治療の安全性・有効性に問題が発生した場合
3） 規制当局からの中止勧告があった場合
4） 試験責任医師の開発方針に変更があった場合
5） その他試験の一部または全体を中止または中断する必要があるような状況が発生した場合

・試験の中止・中断の手順

（1） 試験が何らかの理由により中止または中断された場合、試験責任医師は被験者に速やかにその旨通知し、被験者に対する適切な治療への変更等の適切な処理を行うことを保証する。

（2）　試験責任医師または分担医師が当該医療機関での試験を中止・中断した場合には、医療機関の長に速やかにその旨を文書で報告する。

（3）　試験責任医師が試験を中止・中断した場合、その旨と理由の詳細を医療機関の長に速やかに文書で報告する。

12. 試験実施期間

西暦 2017 年 7 月 15 日から西暦 2018 年 6 月 15 日（登録締切 2018 年 5 月 15 日）

登録期間：11 か月。総研究期間：12 か月。

13. 解析対象（有効性評価の部分集団）、および統計解析方法（主要解析・副次的解析）

本試験の統計解析計画の概要を以下にまとめた。なお、統計解析計画の詳細は、統計解析計画書に記載する。統計解析計画書において本試験実施計画書の概要を修正することがあるが、主要評価項目の定義や解析方法が変更される場合には、本試験実施計画書を改訂する。

1. 解析対象集団

（1）　最大の解析対象集団 (full analysis set：FAS)

本試験に登録され、安全性および有効性データがあるすべての研究対象者を最大の解析対象集団 (FAS) とする。ただし、ベースラインのデータが取得できない研究対象者及び、重大な試験実施計画書違反 (同意未取得、契約期間外の登録等) の研究対象者については除外する。

（2）試験実施計画書に適合した対象集団 (per protocol set：PPS)

FAS から、試験方法や併用療法など試験実施計画書の規定に対して、以下の重大な違反があった症例を除いた研究対象者とする。

選択基準違反

除外基準違反

併用禁止薬違反

併用禁止療法違反

（3）安全性解析対象集団

本試験に登録され、少しでも紫外線照射が行われた被験者を解析の対象とする。

2. 目標症例数および設定根拠

目標症例数　20 例

患者を対象とした新たな臨床試験を適切にデザインするための情報を収集するため、本試験は探索的に実施する。このため有効性・安全性の指標を広く収集するために目標症例数を設定した。理論上、222nmUVC が照射部に紅斑を起こさない可能性がほぼ100%であると想定している。本試験は探索的な位置付けで実施するものであるが、全

症例において紅斑が出現しない場合の片側 95%信頼区間下限が 75%を上回ることが観察できれば、本治療法の安全性に関する判断が可能になると考える。本試験では 2 例から段階的に組み入れ症例を増やし､最終的に 20 例を解析対象症例とする予定である。全 20 例で紅斑が出現しない場合、片側 95%信頼区間下限を求めると 83.9%となる。なお本試験は探索的な位置付けで実施するものであるため、次相の試験の実施を検討する際には、主要評価項目および副次評価項目を総合的に判断することになる。

3. 統計解析項目および解析計画

全ての症例において放射線照射および観察・検体の採取が終了し、データが固定された後に解析を行う。全ての有効性評価において、最大の解析対象集団 (FAS) における解析を主解析とし、参考として試験実施計画書に合致した解析対象集団 (PPS) における解析を行う。安全性の解析は、安全性解析対象集団における解析を実施する。

（1）安全性の解析

1）紅斑の有無

照射量ごとの紅斑非発生割合とその 95%信頼区間を算出する。

2）DNA 損傷の検討

紫外線照射群および非照射群における CPD 生成量を paired t-test を用いて比較検討する。照射群において非照射群に比し有意に CPD 生成量が高値の場合、紫外線照射による DNA 損傷ありと判定する。仮説検定の有意水準は両側 5%とし、信頼区間は両側 95%信頼区間を算出する。

3）有害事象の発生頻度

評価項目について集計表を作成し、割合の推定には 2 項分布の正確な両側 95%信頼区間を群ごとに算出する。必要に応じて Fisher の直接確率計算法を用いて群間比較を行う。

（2）有効性の解析

1）主たる解析

皮膚スワブによる細菌検出頻度を χ 二乗検定を用いて比較検討する。仮説検定の有意水準は両側 5%とし、信頼区間は両側 95%信頼区間を算出する。

2）副次解析

主たる解析結果を補足する考察を行う目的で有効性の副次評価項目の解析を行う。有効性の副次評価項目の解析では多重性の調整は行わない。仮説検定の有意水準は両側 5%とし、信頼区間は両側 95%信頼区間を算出する。

（3）中間解析

本試験において中間解析は行わない。

（4）独立データモニタリング委員会

本試験では独立データモニタリング委員会を設置しない。

（5）最終解析

追跡期間終了後、データが得られ症例が固定された後に解析を行う。統計解析責任者が「解析報告書」をまとめ、試験調整医師及び研究責任者に提出する。

14. 臨床試験実施のための品質保証

1. 品質管理

本試験が安全に、かつ実施計画書に従って実施されているか、データが正確に集積されているかを定期的に確認する目的でモニタリングを行う。

（1） モニタリング責任者及びモニターの指名

研究責任者は、当該研究のモニタリング責任者及びモニターを指名する。

（2） モニタリングの実施

1） 症例に関するモニタリング

モニターは、研究実施中に症例報告書（集積データベース）の確認を行う。また、必要に応じて、神戸大学医学部附属病院で、原資料等（同意書・症例報告書等）の直接閲覧や Off Site モニタリングを行う。モニタリングで確認する項目は「モニタリングの実施に関する手順書」に定める。

2） 症例以外のモニタリング

モニターは開始前、実施中、試験終了（中止・中断）後にモニタリングを実施する。モニタリングで確認する項目は、「モニタリングの実施に関する手順書」に定める。

2. 品質保証

本試験は少数例で実施する探索的な試験であることから、監査は実施しない予定である。

15. 研究対象者の人権および安全性・不利益に対する配慮

1）人権への配慮（個人情報の保護）

症例報告書の作成、被験者のデータの取り扱い等については、被験者のプライバシーの保護に配慮する。すなわち、被験者の氏名やイニシャルは使用せず、被験者識別コードで特定するものとする。研究責任医師は、カルテ番号、患者氏名、および生年月日と、施設にて設定した患者識別コードの対応表（被験者識別コード表）を作成する。被験者識別コード表は、研究責任医師が原本を保管する。

2）安全性・不利益への配慮

本研究の実施に伴い、研究対象者に健康被害が発生した場合は、研究担当者は適切な処置と治療をもって対応する。また、臨床研究を安全に実施する上で必要な情報を収集し検討し、必要に応じて研究計画を変更することを記載する。

16. 研究対象者の費用負担

1） 健康被害の補償

本研究の実施に伴い、研究対象者に健康被害が発生した場合は、研究担当者は適切な処置と治療をもって対応する。また、健康被害に対する補償は、「人を対象とする医学系研究に関する倫理指針」に従って行う。すなわち、本研究に起因して発生した死亡又は後遺障害（障害等級一級から十四級）に対応するために、臨床研究に係る損害保険に加入する。これ以外の健康被害に対しては、研究対象者の保険診療内で検査や治療等、必要な処置を行う。

2）賠償責任保険への加入

賠償責任に備え、研究責任者または研究分担者は賠償責任保険に加入する。

17. 倫理指針およびヘルシンキ宣言等への対応

本試験は人を対象とする医学系研究に関する倫理指針（厚生労働省告示、平成 26 年 12 月 22 日）、およびヘルシンキ宣言、当院の利益相反マネジメント指針を遵守して実施する。

18. 記録文書等の保存

本試験では、「神戸大学大学院医学研究科等における研究データ等の保存期間等に関するガイドライン」に基づき、研究実施に係わる重要な文書（倫理審査委員会への申請書類の控え、研究機関の長からの通知文書、各種申請書・報告書の控、情報公開文書、その他、データ修正履歴、ノートへの記載など研究に用いられる情報の裏付けとなる資料または記録等）を、研究の中止または終了後 10 年間、論文等の研究結果の公表日から 10 年間のいずれか遅い期日まで施錠可能な場所で保存し、その後は個人を特定できない状態にして廃棄する。

各協力研究機関においては研究データ等の保管期間は各協力研究機関の取り決めに従い、適切に保管する。

19. 研究計画の登録

医学雑誌編集者国際委員会（International Committee of Medical Journal Editors: ICMJE）の勧告に基づき、本研究開始までに大学病院医療情報ネットワーク臨床試験登録システム（University hospital Medical Information Network-Clinical Trial Registry: UMIN-CTR）に登録済である（UMIN ID: 000027449）。

20. 研究組織

1．研究責任者および研究分担者

（氏名）	（診療科）	（職名）	（連絡先）
・神戸大学医学部附属病院			
○新倉　隆宏	整形外科	講師	078-382-5985
黒田 良祐	整形外科	教授	078-382-5985
大江 啓介	整形外科	特定助教	078-382-5985
福井 友章	整形外科	医員	078-382-5985
隈部 洋平	整形外科	大学院生	078-382-5985
小田 崇弘	整形外科	大学院生	078-382-5985

（○印：研究責任者）

・共同研究機関：ウシオ電機株式会社

研究責任者：五十嵐　龍志　　新事業推進部、プロジェクトリーダー、フェロー

住所：〒100-8150 東京都千代田区大手町 2-6-1

連絡先：03-6328-3447

・業務委託機関：コスモ・バイオ株式会社

住所：〒135-0016 東京都江東区東陽 2-2-20

連絡先：03-5632-9615

＜研究事務局＞

神戸大学　整形外科　医局

新倉隆宏（研究事務局代表）、大江啓介、福井友章

〒650-0017 兵庫県神戸市中央区楠町 7-5-2

電話：078-382-5985、FAX：078-351-6944

E-mail：tniikura@med.kobe-u.ac.jp（新倉隆宏）

２．プロトコール作成責任者および担当者

責任者：新倉 隆宏　　整形外科　　講師

担当者：福井 友章　　整形外科　　医員

３．データ管理責任者および担当者

責任者：新倉 隆宏　　整形外科　　講師

担当者：福井 友章　　整形外科　　医員

４．モニタリング責任者

西本華子　リハビリテーション部　医員

21. 研究資金源および各研究者の COI 状態の開示

本研究の計画、実施、発表に関して、COI 関係になる企業は存在するが、研究結果に不当な影響を受けることのないよう管理されている。本研究は自主臨床研究であり、研究責任者が所属する診療科の研究費で賄われが、一部はウシオ電機株式会社と神戸大学との契約に基づき、ウシオ電機株式会社より紫外線照射機器の提供と一部資金の拠出を受けて実施される。

22. 実施計画書等の変更

実施計画書や説明文書･同意文書の変更（改訂）は、予め各医療機関の倫理委員会等の承認を得た上で行うこととする。

23. 研究結果の公表

本研究の内容は試験終了後にしかるべき国際・国内学会、peer reviewed journal を選んで発表する。

24. 文献リスト・参考資料

1. 炭山嘉伸、有馬陽一：外科感染制御の現状と問題点。外科　67：125-131、2005.

2. Skaramm I, et al. : Surgical Site Infections in Orthopaedic Surgery Demonstrate Clones Similar to Those in Orthopaedic Staphylococcus aureus Nasal Carriers. J Bone Joint Surg Am. 2014 Jun 4;96（11）:882-888.

3. 西川勝則ら：Surgical Site Infection の感染源は -術野汚染が SSI（創部感染）に及ぼす影響についての検討-。日消外会誌　41（1）：12-21、2008.

4. Wenzel RP, Perl TM：The significance of nasal carriage of Staphylococcus aureus and the incidence of postoperative wound infection. J Hosp Infect. 31（1）：13-24、1995.

5. KreuschS, Schwedler S, Tautkus B, CummeG A, Horn A. UV measurements in microplates suitable for high-throughput protein determination. Anal Biochem. 313:208-15, 2003.

6. Buonanno M et al.,Germicidal Efficacy and Mammalian Skin Safety of 222-nm UV Light, Radiat Res. 2017 Feb 22 [Epub ahead of print]

7. K. Narita, A. Nakane : Graduate school of medicine, at Hirosaki University、Y. Morimoto, T. Igarashi : Ushio Inc. M. Hamblin, T. Dai : Wellman Center for Photomedicine, at Massachusetts General Hospital , Insight into disinfection effect and healing process by irradiating 222 nm UVC light on drug resistant bacteria MRSA infected with Mouse wounds. An article under submission to Journal of Photochemistry & Photobiology, B: Biology.

8. 五十嵐龍志　ウシオ電機　私信　2017 年 3 月 1 日

9. Woods JA, et al. The effect of 222-nm UVC phototesting on healthy volunteer skin: a pilot study. Photodermatol Photoimmunol Photomed. 31（3）:159-66, 2015.
